# Use of a *Candida albicans* SC5314 PacBio HiFi reads dataset to close gaps in the reference genome assembly, reveal a subtelomeric gene family, and produce accurate phased allelic sequences

**DOI:** 10.3389/fcimb.2024.1329438

**Published:** 2024-02-01

**Authors:** Lois L. Hoyer, Brian A. Freeman, Elizabeth K. Hogan, Alvaro G. Hernandez

**Affiliations:** ^1^ Department of Pathobiology, College of Veterinary Medicine, University of Illinois Urbana-Champaign, Urbana, IL, United States; ^2^ Department of Mathematics and Computational Sciences, Millikin University, Decatur, IL, United States; ^3^ Roy J. Carver Biotechnology Center, University of Illinois Urbana-Champaign, Urbana, IL, United States

**Keywords:** genome sequence, *Candida albicans*, pathogenic yeast genomes, PacBio sequence data, allelic sequences, telomere-to-telomere

## Abstract

*Candida albicans* SC5314 is the most-often used strain for molecular manipulation of the species. The SC5314 reference genome sequence is the result of considerable effort from many scientists and has advanced research into fungal biology and pathogenesis. Although the resource is highly developed and presented in a phased diploid format, the sequence includes gaps and does not extend to the telomeres on its eight chromosome pairs. Accurate SC5314 genome assembly is complicated by the presence of extensive repeated sequences and considerable allelic length variation at some loci. Advances in genome sequencing technology provide the tools to obtain highly accurate long-read data that span even the most-difficult-to-assemble genome regions. Here, we describe derivation of a PacBio HiFi data set and creation of a collapsed haploid telomere-to-telomere assembly of the SC5314 genome (ASM3268872v1) that revealed previously unknown features of the strain. ASM3268872v1 subtelomeric distances were up to 19 kb larger than in the reference genome and revealed a family of highly conserved DNA helicase-encoding genes at 10 of the 16 chromosome ends. We also describe alignments of individual HiFi reads to deduce accurate diploid sequences for the most notoriously difficult-to-assemble *C. albicans* genes: the agglutinin-like sequence (*ALS*) gene family. We provide a tutorial that demonstrates how the HiFi reads can be visualized to explore any region of interest. Availability of the HiFi reads data set and the ASM3268872v1 comparative guide assembly will streamline research efforts because accurate diploid sequences can be derived using simple *in silico* methods rather than time-consuming laboratory-bench approaches.

## Introduction

The availability of a genome sequence provides a considerable advantage toward understanding the biology of a species. Strain SC5314 is the most-frequently used background for *Candida albicans* genetic manipulation. Genomic information streamlines gene deletion experiments by facilitating creation and targeting of disruption constructs. However, in the diploid *C. albicans*, these efforts are sometimes complicated by allelic variation (Kim et al., 2022).

A diploid genome assembly for *Candida albicans* strain SC5314 was announced nearly 20 years ago (Jones et al., 2004). Since that time, considerable effort from many scientists has been dedicated toward improvement of this key resource (van het Hoog et al., 2007; Butler et al., 2009; Muzzey et al., 2013). Sequence read data were derived using available technologies including Sanger, 454, and Illumina approaches for genomic fragments, cloned constructs, and PCR-amplified regions. The *Candida* Genome Database (Skrzypek et al., 2017; www.candidagenome.org) is the home for the diploid reference assembly for strain SC5314. The most-current SC5314 sequence available on the National Center for Biotechnology Information (NCBI) website is a haploid representation (i.e. the “A” chromosomes from the *Candida* Genome Database sequence) called ASM18296v3.

Examination of the available *C. albicans* SC5314 assemblies revealed gaps and missing information that remain to be resolved. Regions known to have extreme allelic variability, such as the agglutinin-like sequence (*ALS*) loci, are filled with largely identical sequences across the phased chromosome pairs (Zhao et al., 2003). Ends of chromosomes do not have telomeric repeats despite the repeat sequence being well-established (McEachern and Blackburn, 1994; Butler et al., 2009). These issues are understandable given the read-length limitation (i.e. < 1 kb) of previous DNA sequencing technologies, as well as the potential for rearrangements of repeat-rich cloned DNA.

Advances in DNA sequencing technology now support generation of highly accurate long-read data sets, such as those developed with PacBio HiFi technology (Eid et al., 2009; https://www.pacb.com/technology/hifi-sequencing/how-it-works). Long reads (i.e. 20 kb or more) can span problematic regions in the *C. albicans* genome and derive accurate sequences for highly divergent alleles. Long-read sequence data also provide the opportunity to close the gaps and complete the missing information in the *C. albicans* SC5314 genome assembly, contributing value to this highly used research resource.

Here, we describe derivation of a PacBio HiFi reads data set for *C. albicans* SC5314. The data set readily produced a telomere-to-telomere collapsed haploid genome assembly that revealed previously unrecognized features of this strain. This brief report introduces these new features, but more importantly, provides detailed methods for using the data set to facilitate the study of any gene or chromosomal region of interest.

## Method

### Strain and karyotype

*C. albicans* strain SC5314 was purchased from the American Type Culture Collection (Manassas, VA). Routine culture was conducted in YPD medium (per liter: 10 g yeast extract, 20 g Bacto peptone, 20 g dextrose). Hoyer (2023a) described the method for generating karyotypes using clamped homogeneous electric field (CHEF) electrophoresis.

### Genome reference files

The haploid *C. albicans* SC5314 reference sequence (ASM18296v3; GCF_000182965.3) was downloaded from the National Center for Biotechnology Information (NCBI) website on October 10, 2023 (https://www.ncbi.nlm.nih.gov/datasets/genome/GCF_000182965.3). The complete *C. albicans* SC5314 diploid genome assembly was downloaded from the *Candida* Genome Database (https://www.candidatgenome.org/download/sequence/C_albicans_SC5314) on September 17, 2023 (C_albicans_SC5314_version_A22-s07-m01-r191_chromosomes).

### Derivation of the HiFi reads dataset and assembly of the *C. albicans* SC5314 genome

An isolated colony from a YPD agar plate of *C. albicans* SC5314 was used to inoculate a 20-ml YPD liquid culture in a 50-ml sterile Erlenmeyer flask. The culture was incubated for 16 h at 30°C and 200 rpm shaking. Cells were harvested by centrifugation. Hoyer (2023b) described the protocol for isolating high-molecular-weight DNA for long-read DNA sequencing.

Genomic DNA > 50 kb was sheared with a Megaruptor 3 system (Diagenode) to an average length of 13 kb. A BluePippin system with a 0.75% gel cassette and DNA marker S1 (Sage Science) was used to select fragments of 3-50 kb. These fragments were converted to a sequencing library using the SMRTbell Express Template Prep Kit 3.0 (Pacific Biosciences). The library was sequenced on a single-molecule real-time (SMRT) cell 8M on a PacBio Sequel IIe system using a Sequel II Binding Kit 2.2, the circular consensus sequencing (CCS) mode, and a 30-h movie time. SMRT Link v11.0 was used for CCS and demultiplexing analysis (ccs –min-passes 3 –min-rq 0.99). The HiFi reads data set had 324,036 reads with a mean length of 14,905 bp. The data set included over 4.8 billion bp of sequence information; the longest read was 43,883 bp with N_50_ = 15,357 bp. The HiFi reads data set was deposited into the NCBI Sequence Read Archive (SRA) under accession number SRR23724250.

Filtlong (v0.2.1; Wick and Menzel, 2019) was used to select reads of at least 15 kb, then discard the worst 25% of the reads (–min_length 15000 –keep_percent 75). This process reduced the sequence coverage from approximately 330x to 49x. The length-filtered/subsampled reads were assembled with hifiasm v0.16.1 using default parameters (Cheng et al., 2021). The primary contigs from hifiasm were used for further analysis and deposited into NCBI (ASM3268872v1; GCA_032688725.1). gfatools v0.4 was used to convert files between the GFA and fasta formats (Li and van Zwetselaar, 2019). MUMmer v4.0.0beta2 (nucmer; show-coords -rcl; show-diff -f -r) was used to compare the orientation of the primary contigs to scaffolds in ASM18296v3 (Marçais et al., 2018). Seqkit v.2.0.0 (seq -r -p) was used to reverse complement various contigs to ensure that they matched the same general orientation used in the reference assembly (Shen et al., 2016). Seqkit was also used to calculate basic statistics to summarize genome sequence features. BUSCO v5.3.2 was used to evaluate the content of the assembled genome (Seppey et al., 2019).

### Mining HiFi reads to assemble *ALS* alleles

*ALS* gene sequences from the *Candida* Genome Database (https://www.candidagenome.org) were used as BLAST queries to search the newly assembled PacBio genome sequence (BLAST+ v2.13.0 available from https://www.ncbi.nlm.nih.gov/books/NBK131777). Genome locations were noted and used to target more-detailed examination using the Integrative Genomics Viewer (IGV; v2.16.1; https://igv.org; Robinson et al., 2011). Minimap2 v.2.21 (Li, 2018) was used to map the *C. albicans* SC5314 HiFi reads against the new PacBio genome assembly. The output was passed to SAMtools v1.12 (Li et al., 2009) to create and sort the index file required by IGV. Genome regions encoding each *ALS* gene were visualized in IGV and read identification numbers noted. Reads were extracted from the main HiFi reads dataset (SRR23724250) and aligned using Clustal Omega (http://www.ebi.ac.uk/Tools/msa/clustalo; Madeira et al., 2019). SnapGene software (www.snapgene.com) was used for reverse complementation of reads when needed. *ALS* alleles, each with 1 kb of additional upstream and downstream sequence, were assembled from the consensus of the read alignments and deposited in GenBank under accession numbers OR664373 to OR664386.

### RNA-Seq analysis to assess *YRF1* transcription

RNA-Seq datasets from the NCBI Sequence Read Archive (SRA; https://www.ncbi.nlm.nih.gov/sra) were used to explore the relative transcription level of the *C. albicans YRF1* genes. Stranded datasets, with two replicates each, were derived from strain SC5314 grown in YPD (SRR064145, SRR064146) or YPD with serum (SRR064147, SRR064148; Bruno et al., 2010). The *C. albicans* SC5314 reference sequence (ASM18296v3) and GTF file were downloaded from NCBI (https://www.ncbi.nlm.nih.gov/datasets/genome/GCF_000182965.3). The current genome assembly and annotation included one broken *YRF1* ORF on chromosome 3 (C3_00030C, C3_00010C) and one partial *YRF1* ORF on chromosome 4 (C4_07260W). The chromosome 4 feature was deleted from the GTF file. The broken chromosome 3 ORF was replaced with the *YRF1-5L* sequence from ASM3268872v1 and the GTF annotation revised to report its location. These edits directed all RNA-Seq reads to a single locus that had an accurate sequence. Reads were mapped to the revised reference genome using STAR (Dobin and Gingeras, 2016); featureCounts was used for read summarization (Liao et al., 2014). The enolase (*ENO1*; C1_08500C) and actin (*ACT1*; C1_13700W) genes were used for comparison. Relative gene expression levels (read counts) were normalized to gene length (in kb) and total reads in each replication (in millions). The mean and standard deviation were calculated. The same approach and files were used to assess expression of *YRF1*, *ACT1*, and *ENO1* in RNA-Seq datasets that compared a parental control (ERR1276838) to a *sir2/sir2* mutant (ERR1276839; Freire-Benéitez et al., 2016). Since only one data set was available for each strain, results were expressed as the parent:mutant ratio of normalized read counts.

## Results

### Construction of a SC5314 haploid assembly for use as a comparative guide

The hifiasm program assembled the HiFi reads data set into 8 gapless chromosomes that were bounded by telomeric repeats. An unannotated collapsed haploid version of the assembly was deposited into GenBank (ASM3268872v1) as a guide for comparison to the “A” chromosomes of the reference genome sequence (ASM18296v3). The percent complete and single-copy BUSCOs for ASM3268872v1 were 95.3% (fungi_odb10), 95.4% (ascomycota_odb10) and 98.0% (saccharomycetes_orb10).

**Figure 1:**
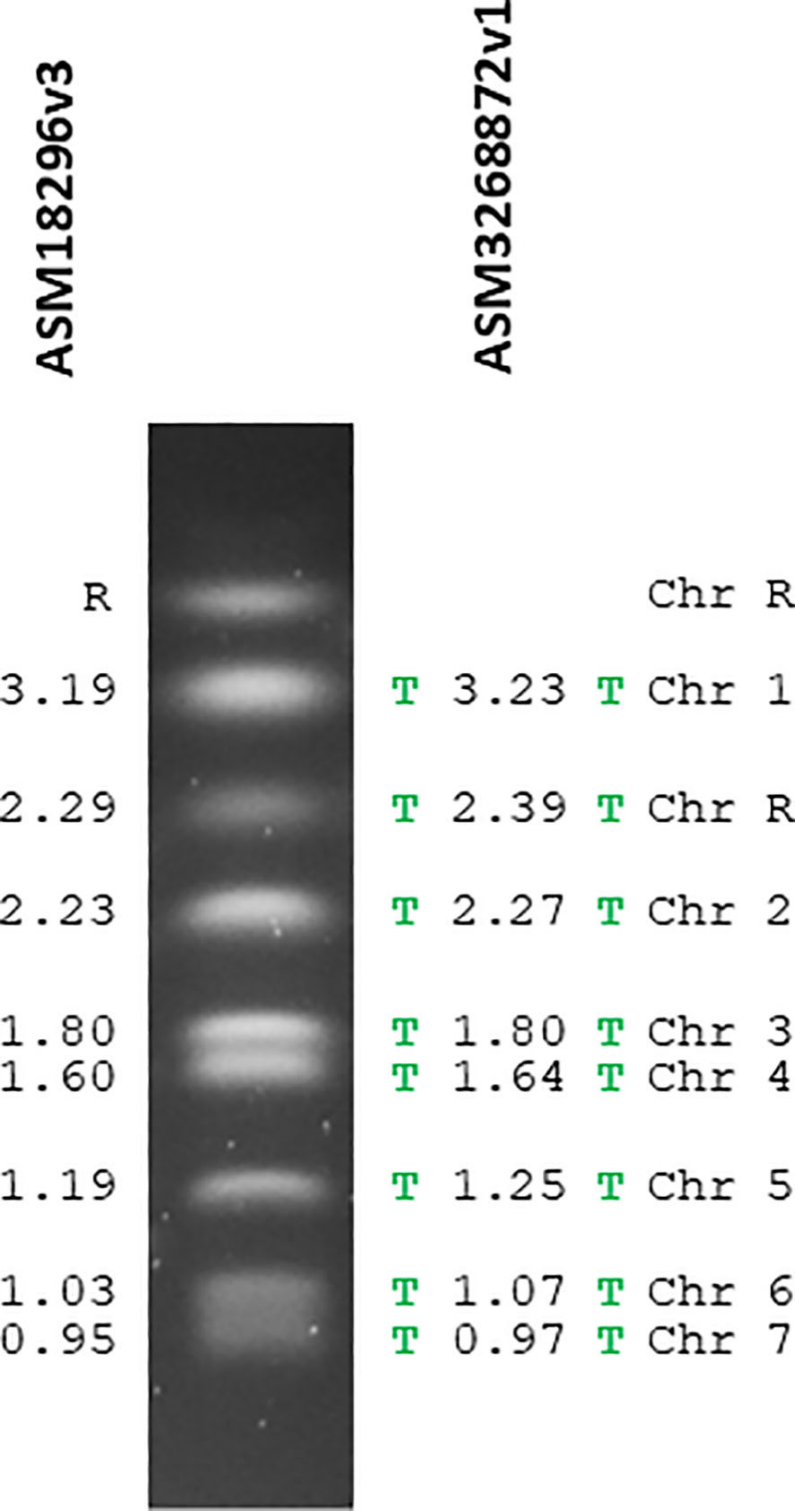
The *C. albicans* SC5314 karyotype annotated with chromosome names and sizes (in Mb) from the reference genome assembly (ASM18296v3; left) and the new PacBio-based assembly (ASM3268872v1; right). Chromosomes labeled “R” encode the tandemly repeated units of ribosomal DNA (rDNA) that expand and contract leading to size heterogeneity (Wickes et al., 1991). Therefore, the size of chromosome R from the genome assembly may not necessarily reflect the karyotype band(s) from a given SC5314 culture flask. The green letter T denotes the presence of telomeric repeat sequences which were located on each chromosome end in the PacBio-based assembly but not in the reference assembly. Interpretation of fragment sizes on the karyotype gel is rooted in use of a *Saccharomyces cerevisiae* strain S288C size standard.

**Table 1** shows the comparative statistics for the reference sequence (ASM18296v3) and the new PacBio assembly (ASM3268872v1). **Figure 1** visualized the genome assemblies in the context of the SC5314 karyotype. The ASM18296v3 reference sequence did not have telomeric repeats on the end of any chromosomes. In contrast, the new assembly had telomeric repeats on each end of every chromosome. The 23-bp telomeric repeats (5’-ACGGATGTCTAACTTCTTGGTGT-3’) were the same as the consensus sequence reported previously (McEachern and Blackburn, 1994; Butler et al., 2009).

**Table 1:** Comparison between *C. albicans* SC5314 reference genome (ASM18296v3) and new PacBio (ASM3268872v1) haploid assemblies.

Feature	ASM18296v3	ASM3268872v1
	GCA_000182965.3	GCA_032688725.1
Sequence length (bp)	14,282,666	14,612,850
Ungapped sequence length (bp)	14,276,407	14,612,850
No. of contigs	88	8
Contig N_50_ (bp)	334,280	2,271,053
No. of scaffolds	8	8
Largest scaffold (bp)	3,188,341	3,230,033
Scaffold N_50_ (bp)	2,231,883	2,271,053
No. of spanned gaps	80	0

### Reconciling length difference estimates between ASM18296v3 and ASM3268872v1

Haploid chromosome sizes were larger for ASM3268872v1 than for the reference assembly (**Figure 1**). For example, chromosome 7 was approximately 22 kb larger in ASM3268872v1 and chromosome 5 was 55 kb larger. One obvious source of length variation was sequences at the chromosome ends. **Supplementary Figure S1** diagrams 20 kb of each ASM3268872v1 subtelomeric region to provide an at-a-glance feature summary. The *TLO* gene family (telomere-associated; Dunn et al., 2022; O’Connor-Moneley et al., 2023) is found in this region. *TLO* genes provided a point of reference since they were originally defined as being no more than 12 kb from a telomere. **Supplementary Figure S1** includes other ORFs that were larger than 1 kb and that were located completely within each 20-kb region. The red-circled X indicated the approximate location where the reference assembly stopped, allowing for easy visualization of the amount of new sequence included in ASM3268872v1. The sequence at both ends of chromosomes 1 and 3 were so different between ASM18296v3 and ASM3268872v1 that the red-circled X could not be placed. These large sequence differences were denoted by an unequal sign.

The reference assembly extended nearly to the telomere on some chromosomes (e.g. chromosome R Left) while nearly 20 kb of sequence was not known for others such as chromosome 5 Right and chromosome 7 Left (**Supplementary Figure S1**). In the PacBio-based assembly, subtelomeric sequences for chromosome 7 accounted for potentially all the 22 kb length variation compared to the reference assembly. For chromosome 5, the new subtelomeric sequences accounted for approximately half of the 55-kb difference.

Another potential source of length variation for the chromosomes was filling 80 sequence gaps (i.e. regions of NNNN) with the new PacBio long-read data. Chromosome 1 from the reference assembly was used to explore the nature of the gaps and how they were resolved in the new assembly. In some cases, gap sizes predicted in the reference assembly were filled by approximately the same number of nucleotides in ASM3268872v1. One example was a 41-bp gap at position 1,291,696 in the reference sequence that was replaced by 38 bp in the new assembly. In other cases, closing a gap led to a larger size difference between the assemblies. One example was the 116 N inserted at 1,337,290 in the reference sequence, 317 bp upstream of the start codon for C1_06320W. In the new assembly, this gap was closed and the size of the region reduced to 172 bp. Chromosome 1 in the reference assembly also had several instances of gaps that broke an open reading frame. For example, 25 N were inserted at the end of C1_06690W (*CYK3*) resulting in a 933-bp ORF. In the new assembly, the ORF was 3063 bp. Another example was C1_12490W that was extended from 4404 bp to 4761 bp when a gap upstream of the start codon was repaired. The total of these instances will account for the majority of the remaining length estimate differences between the haploid assemblies (**Figure 1**). The PacBio long-read data set can be used to resolve any of these genomic regions into accurate diploid alleles as demonstrated below.

### A subtelomeric gene family encoding highly conserved DNA helicases

ASM3268872v1 revealed a previously unrecognized subtelomeric gene family on 10 of the 16 chromosome ends. Two locations in the reference assembly had fragments of these genes. One location was annotated as two separate ORFs on the left end of chromosome 3 (C3_00030C and C3_00010C); the other was a 357-bp fragment of the start of the gene that ran off the right end of chromosome 4 (C4_07260W). Perhaps ironically, in the new PacBio-based assembly, chromosome 3 was one of the few locations that did not have a copy of the gene.

Each of the genes in the newly revealed subtelomeric family were 4269 bp, encoding a 1426 amino acid protein. Alignment of the sequences revealed 99.6 to 100% identity at the nucleotide level and 99.3 to 100% identity for the amino acids. The proteins had DEAD-box helicase and ATP-binding motifs. ORF C3_00030C in the reference genome was annotated as *YRF1* in the *Candida* Genome Database because it has similarity to these functional domains in the *S. cerevisiae YRF1* alleles (Yamada et al., 1998). *S. cerevisiae* has 8 *YRF1* alleles (*YRF1-1* through *YRF1-8*), each located in the subtelomeric region of 7 of the 32 chromosome arms (Chr IV, Right; ChrV, Right; Chr VII, Right; two loci in tandem on Chr XII, Right; Chr XIV, Left; Chr XVI, Left; and Chr XV, Right; https://www.yeastgenome.org). In each instance, the ORF is transcribed toward the telomere, just as for the genes in *C. albicans*. Comparisons between the *S. cerevisiae YRF1* alleles showed that they are 99.1 to 100% identical in nucleotide sequence, despite length variation that ranges from 4149 bp to 5580 bp. Alignment of the sequences showed the potential for start codons in the longer genes that are apparently not in-frame for the shorter ones (**Supplementary Figure S2**). Alignment between the *C. albicans* and *S. cerevisiae* genes showed approximately 45% identity that was confined to the region encoding the DEAD-box and ATP-binding domains. The *YRF1* name was retained for these newly recognized subtelomeric coding regions in *C. albicans*, with an allelic designation added to indicate the location of each. For example, *YRF1-1R* was found on the Right arm of *C. albicans* chromosome 1 (**Supplementary Figure S1**).

RNA-Seq data from the SRA were examined to determine if *YRF1* alleles were transcribed. Datasets recording stranded reads during growth in YPD and in YPD + serum were selected (see Method). RNA-Seq reads were mapped and counted using the ASM18296v3 GTF file that was edited to include only one full copy of the *YRF1* gene (see Method). In YPD medium, *YRF1* had 51 ± 5 reads, compared to 1213 ± 45 for *ACT1* and 4039 ± 788 for *ENO1*. Similar values were observed for growth in YPD + serum: 45 ± 4 reads for *YRF1*, 1504 ± 84 for *ACT1*, and 4182 ± 355 for *ENO1*. These data demonstrated that *YRF1* was transcribed, but at a level considerably lower than genes that are highly active during *in vitro* growth. Using a *sir2/sir2* strain, Freire-Benéitez et al. (2016) demonstrated that telomeric heterochromatin silences gene expression in *C. albicans* subtelomeric regions. Analysis of their RNA-Seq datasets (ERR1276838 control, ERR1276839 *sir2/sir2*) with our edited GTF file produced a ratio of 2.0 for *YRF1* expression in the *sir2/sir2* strain compared to its wild-type parent. Ratios were 0.9 for *ACT1* and 1.8 for *ENO1*. These results were consistent with the published report and suggested that *YRF1* alleles were silenced by telomeric heterochromatin.

### Use of the PacBio HiFi reads data set to produce accurate phased diploid allelic sequences

The most-highly desired *C. albicans* SC5314 genome sequence would be presented in a telomere-to-telomere phased diploid format; to date, such a resource has not been delivered. To test whether the PacBio HiFi reads data set has the potential to serve as the basis for such a result, we used the data set to evaluate the most notoriously difficult-to-assemble genes: the agglutinin-like sequence (*ALS*) family (Hoyer et al., 2008). *ALS* genes encode large glycoproteins that are involved in *C. albicans* adhesion to host cells, to each other, to abiotic surfaces, and to other microbes (reviewed in Hoyer and Cota, 2016).

**Figure 2:**
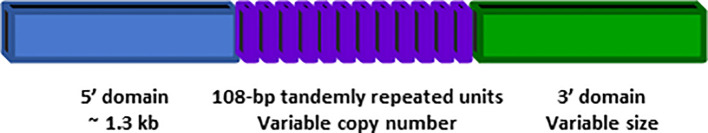
Basic organization of *C. albicans* agglutinin-like sequence (*ALS*) genes. The simplest summary of *ALS* gene structure includes a 5’ domain that encodes the adhesive and aggregative functions of the proteins (Lin et al., 2014), a central domain of variable copy numbers of a 108-bp tandemly repeated motif, and a 3’ domain of variable length and sequence (Hoyer et al., 2008). Some *ALS* genes are highly conserved in the 5’ domain sequence (e.g. *ALS1*, *ALS3*, *ALS5*) and others are conserved in the 3’ domain sequence (*ALS5*, *ALS6*). The copy number of 108-bp repeated motifs can be quite high with the center of an *ALS* gene consisting of several kb of essentially identical sequences. Computational assembly of data from Sanger, 454, and Illumina technologies failed in the face of these challenges. Sequencing of cloned fragments was also problematic because repeat regions tended to rearrange when produced in *Escherichia coli*. In contrast, PacBio HiFi reads can span the full length of an *ALS* gene and assign it to the appropriate genomic location using unique adjacent loci. The PacBio HiFi reads dataset featured mean read length of approximately 15 kb; reads of at least 15 kb were used to create the collapsed haploid assembly (ASM3268872v1). Moreover, the HiFi read lengths provided an accurate genomic context and sequence for sometimes-extreme allelic variants in the *ALS* family, placing them onto a specific chromosome haplotype. The PacBio HiFi reads data set described here revealed the allelic sequences of the *ALS* family for the first time. This approach to deducing allelic sequences can be applied to any region of strain SC5314 by mining the HiFi reads data set (SRR23724250) as demonstrated in **Supplementary File S1**.

The schematic of *ALS* gene organization (**Figure 2**) illustrates why these ORFs have been so difficult to assemble using data from sequence technologies with shorter read lengths. Each *ALS* gene has a 5’ region that encodes the peptide-binding cavity, a central domain of highly conserved copies of a tandemly repeated 108-bp motif, and a 3’ end of more-diverse sequence that encodes a Ser/Thr-rich portion of the protein that is destined for heavy modification with carbohydrate (Kapteyn et al., 2000). *ALS* genes occupy eight different physical locations on three of the *C. albicans* chromosomes (chromosomes 6, 3, and R). Sequences for some 5’ domain and 3’ domain regions are nearly identical at more than one *ALS* locus. However, the largest obstacle for accurate *ALS* gene assembly is the tandem-repeat region. The 108-bp repeated motif is highly conserved and, in some *ALS* genes, can be present in dozens of copies that span several kb of length. Shorter sequence reads may not have sufficient length to anchor their information into a unique genome location, resulting in broken assemblies.

Neither the current phased diploid SC5314 assembly on the *Candida* Genome Database nor the new PacBio-based haploid assembly (ASM3268872v1) presented accurate sequences for the *ALS* loci. On the *Candida* Genome Database, sequences at the *ALS* loci were identical between the alleles despite considerable evidence that most *ALS* alleles are heterogeneous in length in strain SC5314 (Hoyer et al., 1995; Zhao et al., 2003; Zhao et al., 2004; Zhao et al., 2007b). The new haploid PacBio-based assembly had broken genes at most of the *ALS* loci, presumably from attempting to reconcile sometimes-extreme allelic length differences within the data.

To deduce *ALS* allelic sequences from the HiFi reads data set, we used the ASM3268872v1 assembly as a guide to find the *ALS* loci, visualized the region using the Integrative Genomics Viewer (IGV), extracted individual long reads (approximately 18-25 kb), and aligned them to record a consensus sequence. Sequence mismatches among the reads tended to be one-nucleotide insertions or deletions, most frequently occurring in areas of repeated nucleotides (e.g. AAAA instead of AAA). Alignment of 5-6 individual long reads for each *ALS* allele was sufficient to develop a robust consensus sequence. This process is visualized in **Supplementary Figure S3**. **Supplementary File S1** offers the analysis programs and commands used to generate this figure with the goal of enabling novice users to locate genes of interest and deduce their allelic sequences from the PacBio HiFi reads data set.

### Comparing PacBio-derived *ALS* allelic sequences to results from previous methods

The idea that PacBio long-read sequence technology may have finally delivered accurate *ALS* sequences for strain SC5314 prompted comparisons between the newly assembled alleles and those previously reported for the strain. **Table 2** summarizes the *ALS* alleles assembled from the PacBio HiFi reads data set and their GenBank accession numbers. GenBank deposits included 1 kb of upstream and downstream sequence for each *ALS* allele. **Table 2** also lists all available versions of the SC5314 full-length *ALS* genes/alleles from GenBank. Alleles described by Muzzey et al. (2013) were incorporated into the phased diploid SC5314 assembly (i.e. chromosomes “A” and “B”) reported on the *Candida* Genome Database.

**Table 2:** Comparison among *C. albicans* SC5314 *ALS* gene sequences available in public databases.

Gene	ID	Source	Size*	Reference
***ALS1-1***	**OR664373**	**GenBank**	**4971 (21)**	**This study**
***ALS1-2***	**OR664374**	**GenBank**	**3783 (10)**	**This study**
*ALS1*	XM_712984.2	GenBank	3783	Muzzey et al., 2013
*ALS1_A*	C6_03700W_A	*Candida* Genome Database	3783	
*ALS1_B*	C6_03700W_B	*Candida* Genome Database	3783	

***ALS2-1***	**OR664375**	**GenBank**	**6624 (39)**	**This study**
***ALS2-2***	**OR664376**	**GenBank**	**6081 (34)**	**This study**
*ALS2*	XM_707553	GenBank	7089	Muzzey et al., 2013
*ALS2_A*	C6_04380W_A	*Candida* Genome Database	7089	
*ALS2_B*	C6_04380W_B	*Candida* Genome Database	7089	

***ALS3-1***	**OR664377**	**GenBank**	**3474 (12)**	**This study**
***ALS3-2***	**OR664378**	**GenBank**	**3144 (9)**	**This study**
*ALS3-1*	AY223552	GenBank	3468	Zhao et al., 2004
*ALS3-2*	AY223551	GenBank	3144	Zhao et al., 2004
*ALS3*	XM_705343.2	GenBank	3468	Muzzey et al., 2013
*ALS3_A*	CR_07070C_A	*Candida* Genome Database	3468	
*ALS3_B*	CR_07070C_B	*Candida* Genome Database	3468	

***ALS4-1***	**OR664379**	**GenBank**	**6300 (36)**	**This study**
***ALS4-2***	**OR664380**	**GenBank**	**3819 (13)**	**This study**
*ALS4*	XM_705333.2	GenBank	6303	Muzzey et al., 2013
*ALS4_A*	C6_04130C_A	*Candida* Genome Database	6303	
*ALS4_B*	C6_04130C_B	*Candida* Genome Database	6303	

***ALS5-1***	**OR664381**	**GenBank**	**4152 (5)**	**This study**
***ALS5-2***	**OR664382**	**GenBank**	**4044 (4)**	**This study**
*ALS5-1*	AY227440	GenBank	4152	Zhao et al., 2003
*ALS5-2*	AY227439	GenBank	4044	Zhao et al., 2003
*ALS5*	XM_712981.2	GenBank	4044	Muzzey et al., 2013
*ALS5_A*	C6_03690W_A	*Candida* Genome Database	4044	
*ALS5_B*	C6_03690W_B	*Candida* Genome Database	4044	

***ALS6***	**OR664383**	**GenBank**	**4101 (4)**	**This study**
*ALS6*	AY225310	GenBank	4101	Zhao et al., 2007a
*ALS6*	XM_710986	GenBank	4101	Muzzey et al., 2013
*ALS6-2*	EU444081	GenBank	4101	Unpublished
*ALS6_A*	C3_06190C_A	*Candida* Genome Database	4101	
*ALS6_B*	C3_06190C_B	*Candida* Genome Database	4101	

***ALS7***	**OR664384**	**GenBank**	**6003 (15)**	**This study**
*ALS7*	XM_710972.2	GenBank	4707	Muzzey et al., 2013
*ALS7_A*	C3_06320W_A	*Candida* Genome Database	4707	
*ALS7_B*	C3_06320W_B	*Candida* Genome Database	4707	

***ALS9-1***	**OR664385**	**GenBank**	**5673 (19)**	**This study**
***ALS9-2***	**OR664386**	**GenBank**	**5718 (17)**	**This study**
*ALS9-1*	AY269423	GenBank	5565	Zhao et al., 2003
*ALS9-2*	AY269422	GenBank	5502	Zhao et al., 2003
*ALS9*	XM_712985.2	GenBank	5673	Muzzey et al., 2013
*ALS9_A*	C6_03710W_A	*Candida* Genome Database	5673	
*ALS9_B*	C6_03710W_B	*Candida* Genome Database	5673	

*Gene size is listed in bp; copy number of tandem repeat units is shown in parentheses.Bold text in the table refers to sequences that were associated with the current study.

The largest size difference between PacBio-derived *ALS* alleles at any locus was attributable to differences in tandem repeat copy number. For example, *ALS1-1* (OR664373) was 1188 bp larger than *ALS1-2* (OR664374), a difference of 11 copies of the 108-bp tandem repeat sequence (**Table 2**). Other small allelic sequence differences were observed, but not detailed here. For some loci like *ALS6*, gene length was identical among the reported sequences with > 99% sequence identity. *ALS6* assembled into only one allele (100% identity) from the PacBio data, consistent with the conclusion of homozygosity at the *ALS6* locus in strain SC5314 (**Supplementary Figure S3**). The PacBio reads also produced only one allele for *ALS7* (**Table 2**). Homozygosity of a 6003-bp *ALS7* in strain SC5314 was consistent with predictions from agarose gel images published by Zhang et al. (2003). The sequence derived from the PacBio data (OR664384) was approximately 1.3 kb larger than the previous estimate (XM_710972.2), a difference of 12 tandem repeat unit copies.

*ALS5*, *ALS1*, and *ALS9* are contiguous on chromosome 6; sequences from strain SC5314 were derived by cloning and Sanger sequencing PCR products amplified from heterozygous knockout strains (Zhao et al., 2003). Zhao et al. (2003) accurately predicted the sizes of the *ALS5* alleles but did not compile full-length *ALS1* sequences. The *ALS1* sequences reported by Muzzey et al. (2013) represented the smaller *ALS1* allele (**Table 2**). Alleles of *ALS9-1* and *ALS9-2* vary considerably in SC5314 (Zhao et al., 2003). Allelic sizes derived from cloned fragments did not match those from the PacBio reads (**Table 2**). The sequence reported by Muzzey et al. (2013) corresponded to *ALS9-1*.

Full-length sequences for *ALS2* and *ALS4* historically have been difficult to assemble because they have nearly 4 kb of tandem repeat units in the center of the coding region. PacBio data separated the alleles using long reads that anchored each allele into its chromosomal context (**Supplementary Figure S3**). Muzzey et al. (2013) overestimated the size of the *ALS2* alleles, but closely approximated the size of *ALS4-1* (**Table 2**).

*ALS3* alleles from SC5314 have been studied extensively with respect to differential adhesive function (Oh et al., 2005). Zhao et al. (2004) accurately reported the size of *ALS3-2*, but the PacBio reads suggested that *ALS3-1* was 6 bp longer than earlier reports. Some alignments between the newly assembled PacBio alleles and those from earlier reports showed small differences in nucleotide sequences outside of the tandem repeat region. Overall, however, the size of the newly assembled *ALS3* alleles compared favorably with estimates from previous publications that used methods such as PCR amplification or Southern blotting to study strain SC5314 (Hoyer et al., 1998; Zhao et al., 2004).

## Discussion

A PacBio HiFi reads data set for *C. albicans* strain SC5314 assembled readily into 8 gapless chromosome-sized contigs that were bounded by telomeric repeats. The new collapsed haploid assembly, ASM3268872v1, revealed features of the SC5314 genome that were previously not reported. Among these features were the complete subtelomeric regions for each chromosome that revealed a highly conserved family of helicase-encoding genes (*YRF1*). The large size of the *C. albicans YRF1* genes (4269 bp) and the high degree of similarity between subtelomeric sequences on all the chromosomes explain the difficulty in assembling them accurately from historic data that had shorter read lengths (i.e. < 1 kb). Use of PacBio HiFi reads longer than 15 kb to assemble the chromosomes placed each subtelomeric region into its accurate chromosomal context.

PacBio HiFi long reads of 18-25 kb spanned the entire coding region for genes in the *ALS* family, providing the first accurate allelic sequences for each *ALS* locus, as well as upstream and downstream flanking sequences that facilitate construction and targeting of disruption cassettes for genetic manipulation. The methods demonstrated for exploration of the *ALS* family can be applied to any region in the SC5314 genome. Availability of the PacBio HiFi reads data set eliminates the need for costly laboratory-bench approaches to derive accurate diploid sequences at any locus.

Information presented here suggests the possibility that the HiFi reads data set could support construction of an accurate, phased diploid genome assembly for strain SC5314. This resource would be tremendously valuable to the *C. albicans* community. In the meantime, placing the data set into the hands of researchers will ensure its immediate use to resolve and verify allelic variation for any region of interest, advancing our understanding of *C. albicans* biology and pathogenesis.

## Data availability statement

The datasets presented in this study can be found in online repositories. The names of the repository/repositories and accession number(s) can be found in the article/**Supplementary Material**.

## Author contributions

LH: Conceptualization, Data curation, Formal analysis, Funding acquisition, Investigation, Methodology, Project administration, Resources, Supervision, Writing – original draft, Writing – review & editing. BF: Data curation, Formal analysis, Investigation, Writing – review & editing. EH: Investigation, Methodology, Writing – review & editing. AH: Investigation, Methodology, Writing – review & editing.
