# Supplementary material for: Use of a Candida albicans SC5314 PacBio HiFi reads dataset to close gaps in the reference genome assembly, reveal a subtelomeric gene family, and produce accurate phased allelic sequences
Source: Front Cell Infect Microbiol. 2024 Feb 1;14:1329438. doi: 10.3389/fcimb.2024.1329438 (PMC10867151; doi:10.3389/fcimb.2024.1329438)
Supplement: Supplementary file 1 [file DataSheet_1.docx]

CaYrf1-5L MSDHESDNYDSEPDIFEI----IVSDSEDDSDMEDLFSAPATGTQNTTMDEECQVEEGVS 56

Yrf1-4 ------------------------------------------------------------ 0

Yrf1-1 ------------------------------------------------------------ 0

Yrf1-5 ------------------------------------------------------------ 0

Yrf1-8 ------------------------------------------------------------ 0

Yrf1-6 MEIENEQICTCIAQILHLLNSLIITFLDDD------------------------------ 30

Yrf1-7 MEIENEQICTCIAQILHLLNSLIITFLDDD------------------------------ 30

Yrf1-2 ------------------------------------------------------------ 0

Yrf1-3 MEIENEQICTCIAQILHLLNSLIITFLDDD------------------------------ 30

CaYrf1-5L RRRTVEGHHVSEDNEDYLQRTYGVKSTQYCFRLGKKHIYCRNFIIEERSSGNVVI----- 111

Yrf1-4 ------------------------------------------------------------ 0

Yrf1-1 ------------------------------------------------------------ 0

Yrf1-5 ------------------------------------------------------------ 0

Yrf1-8 ------------------------------------------------------------ 0

Yrf1-6 -----------------------------KTETGQSFVYIDGFLVKKHNNQHTIVNFETY 61

Yrf1-7 -----------------------------KTETGQSFVYIDGFLVKKHNNQHTIVNFETY 61

Yrf1-2 ------------------------------------------------------------ 0

Yrf1-3 -----------------------------KTETGQSFVYIDGFLVKKHNNQHTIVNFETY 61

CaYrf1-5L --------------------------------NPTARFYKRASRKVK------------- 126

Yrf1-4 ------------------------------------------------------------ 0

Yrf1-1 ---MKVSDRRKFEKANFDEFESALNNKNDLVHCPSITLFESIPTEVRSFYEDEKSGLIKV 57

Yrf1-5 ---MKVSDRRKFEKANFDEFESALNNKNDLVHCPSITLFESIPTEVRSFYEDEKSGLIKV 57

Yrf1-8 ---MKVSDRRKFEKANFDEFESALNNKNDLVHCPSITLFESIPTEVRSFYEDEKSGLIKV 57

Yrf1-6 KNKMKVSDRRKFEKANFDEFESALNNKNDLVHCPSITLFESIPTEVRSFYEDEKSGLIKV 121

Yrf1-7 KNKMKVSDRRKFEKANFDEFESALNNKNDLVHCPSITLFESIPTEVRSFYEDEKSGLIKV 121

Yrf1-2 ------------------------------------------------------------ 0

Yrf1-3 KNKMKVSDRRKFEKANFDEFESALNNKNDLVHCPSITLFESIPTEVRSFYEDEKSGLIKV 121

CaYrf1-5L -------------------------------------SDLE--SVPVHQFTPDEKEIRV- 146

Yrf1-4 ------------------------------------------------------------ 0

Yrf1-1 VKFRTGAMDRKRSFEKIVISVMVGKNVQKFLTFVEDEPDFQGGPIPSKYLIPKKINLMVY 117

Yrf1-5 VKFRTGAMDRKRSFEKIVISVMVGKNVQKFLTFVEDEPDFQGGPIPSKYLIPKKINLMVY 117

Yrf1-8 VKFRTGAMDRKRSFEKIVISVMVGKNVQKFLTFVEDEPDFQGGPIPSKYLIPKKINLMVY 117

Yrf1-6 VKFRTGAMDRKRSFEKIVVSVMVGKNVQKFLTFVEDEPDFQGGPIPSKYLIPKKINLMVY 181

Yrf1-7 VKFRTGAMDRKRSFEKIVVSVMVGKNVQKFLTFVEDEPDFQGGPIPSKYLIPKKINLMVY 181

Yrf1-2 ---------------------------------------------------------MVY 3

Yrf1-3 VKFRTGAMDRKRSFEKIVVSVMVGKNVQKFLTFVEDEPDFQGGPIPSKYLIPKKINLMVY 181

CaYrf1-5L ------------DDADLYEMVESKEETVRGMHCVSTQACVGIKKH-----VKLYMPREVV 189

Yrf1-4 ------------------------------------------------------------ 0

Yrf1-1 TLFQVHTLKFNRKDYDTLSLFYLNRGYYNELSFRVLERCHEIASARPNDSSTMRTFTDFV 177

Yrf1-5 TLFQVHTLKFNRKDYDTLSLFYLNRGYYNELSFRVLERCHEIASARPNDSSTMRTFTDFV 177

Yrf1-8 TLFQVHTLKFNRKDYDTLSLFYLNRGYYNELSFRVLERCHEIASARPNDSSTMRTFTDFV 177

Yrf1-6 TLFQVHTLKFNRKDYDTLSLFYLNRGYYNELSFRVLERCYEIASARPNDSSTMRTFTDFV 241

Yrf1-7 TLFQVHTLKFNRKDYDTLSLFYLNRGYYNELSFRVLERCYEIASARPNDSSTMRTFTDFV 241

Yrf1-2 TLFQVHTLKFNRKDYDTLSLFYLNRGYYNELSFRVLERCHEKASARPNDSSTMRTFTDFV 63

Yrf1-3 TLFQVHTLKFNRKDYDTLSLFYLNRGYYNELSFRVLERCYEIASARPNDSSTMRTFTDFV 241

CaYrf1-5L EGSNF-----DMKVRDEGYAQVTRSTAVL--D--QFFSVYEFDD---KMSEPDSTAG--- 234

Yrf1-4 ------------------------------------------------------------ 0

Yrf1-1 SGAPIVRSLQKSTIRKYGYNLAPYMFLLLHVDELSIFSAYQASLPGEKKVDTERLKRDLC 237

Yrf1-5 SGAPIVRSLQKSTIRKYGYNLAPYMFLLLHVDELSIFSAYQASLPGEKKVDTERLKRDLC 237

Yrf1-8 SGAPIVRSLQKSTIRKYGYNLAPYMFLLLHVDELSIFSAYQASLPGEKKVDTERLKRDLC 237

Yrf1-6 SGTPIVRGLQKSTIRKYGYNLAPYMFLLLHVDELSIFSAYQASLPGEKKVDTERLKRDLC 301

Yrf1-7 SGTPIVRGLQKSTIRKYGYNLAPYMFLLLHVDELSIFSAYQASLPGEKKVDTERLKRDLC 301

Yrf1-2 SGAPIVRSLQKSTIRKYGYNLAPYMFLLLHVDELSIFSAYQASLPGEKKVDTERLKRDLC 123

Yrf1-3 SGTPIVRGLQKSTIRKYGYNLAPYMFLLLHVDELSIFSAYQASLPGEKKVDTERLKRDLC 301

CaYrf1-5L -YIDAGRKIYKAVYDDVIAQHDLANFLGSSLTYHFR------------------------ 269

Yrf1-4 ------------------------------------------------------------ 0

Yrf1-1 PRKPIEIKYFSQICNDMMNKKD---RLGDILHIILRACALNFGAGPRGGAGDEEDRSITN 294

Yrf1-5 PRKPIEIKYFSQICNDMMNKKD---RLGDILHIILRACALNFGAGPRGGAGDEEDRSITN 294

Yrf1-8 PRKPIEIKYFSQICNDMMNKKD---RLGDILHIILRACALNFGAGPRGGAGDEEDRSITN 294

Yrf1-6 PRKPTEIKYFSQICNDMMNKKD---RLGDILHIILRACALNFGAGPRGGAGDEEDRSITN 358

Yrf1-7 PRKPTEIKYFSQICNDMMNKKD---RLGDILHIILRACALNFGAGPRGGAGDEEDRSITN 358

Yrf1-2 PRKPTEIKYFSQICNDMMNKKD---RLGDILHIILRACALNFGAGPRGGAGDEEDRSITN 180

Yrf1-3 PRKPTEIKYFSQICNDMMNKKD---RLGDILHIILRACALNFGAGPRGGAGDEEDRSITN 358

CaYrf1-5L -----------NERFVRVLAPETVKQYGTCFGRMLSLIMASKDFDLCERVDTSIANELSG 318

Yrf1-4 ------------------------------------------------------------ 0

Yrf1-1 EEPIIPSVDEHGLKVCKLRSPNTPRRLRKTLDAVKALLVSS---CACTARDLDIFDDTNG 351

Yrf1-5 EEPIIPSVDEHGLKVCKLRSPNTPRRLRKTLDAVKALLVSS---CACTARDLDIFDDTNG 351

Yrf1-8 EEPIIPSVDEHGLKVCKLRSPNTPRRLRKTLDAVKALLVSS---CACTARDLDIFDDTNG 351

Yrf1-6 EEPIIPSVDEHGLKVCKLRSPNTPRRLRKTLDAVKALLVSS---CACTARDLDIFDDNNG 415

Yrf1-7 EEPIIPSVDEHGLKVCKLRSPNTPRRLRKTLDAVKALLVSS---CACTARDLDIFDDNNG 415

Yrf1-2 EEPIIPSVDEHGLKVCKLRSPNTPRRLRKTLDAVKALLVSS---CACTARDLDIFDDNNG 237

Yrf1-3 EEPIIPSVDEHGLKVCKLRSPNTPRRLRKTLDAVKALLVSS---CACTARDLDIFDDNNG 415

CaYrf1-5L A-AWEQMINPLLEWIV-------------SQTADSDTLCTML-VRPYLYNFK-------T 356

Yrf1-4 ------------------------------------------------------------ 0

Yrf1-1 VAMWKW-IKILYHEVAQETTLKDSYRITLVPSSDGISVCGKLFNREYVRGFYFACKAQFD 410

Yrf1-5 VAMWKW-IKILYHEVAQETTLKDSYRITLVPSSDGISVCGKLFNREYVRGFYFACKAQFD 410

Yrf1-8 VAMWKW-IKILYHEVAQETTLKDSYRITLVPSSDGISVCGKLFNREYVRGFYFACKAQFD 410

Yrf1-6 VAMWKW-IKILYHEVAQETALKDSYRITLVPSSDGVSVCGKLFNREYVRGFYFACKAQFD 474

Yrf1-7 VAMWKW-IKILYHEVAQETALKDSYRITLVPSSDGVSVCGKLFNREYVRGFYFACKAQFD 474

Yrf1-2 VAMWKW-IKILYHEVAQETALKDSYRITLVPSSDGVSVCGKLFNREYVRGFYFACKAQFD 296

Yrf1-3 VAMWKW-IKILYHEVAQETALKDSYRITLVPSSDGVSVCGKLFNREYVRGFYFACKAQFD 474

CaYrf1-5L KAWQSVAWCLKYIDALKSMFKMAMFLKSRDEFSSFCSQPQEYGCYFYLQTI--YARLVQQ 414

Yrf1-4 -MWKTLGR---------------------------------------VEQLLPYASLILR 20

Yrf1-1 NLWGELNNC------------------------------------FYMPTVVDIASLILR 434

Yrf1-5 NLWGELNNC------------------------------------FYMPTVVDIASLILR 434

Yrf1-8 NLWGELNNC------------------------------------FYMPTVVDIASLILR 434

Yrf1-6 NLWEELNDC------------------------------------FYMPTVVDIASLILR 498

Yrf1-7 NLWEELNDC------------------------------------FYMPTVVDIASLILR 498

Yrf1-2 NLWEELNDC------------------------------------FYMPTVVDIASLILR 320

Yrf1-3 NLWEELNDC------------------------------------FYMPTVVDIASLILR 498

* : : : * *: :

CaYrf1-5L RREVV-EMEVSGIDKR---TVFVREIKVT--TDYISKLYHDGVKLYMSEYGSLCRVMGLE 468

Yrf1-4 NREVLFREPKRGIDEYLENDSFFQMIPVKYREIVLPKLRRDTNK---------------- 64

Yrf1-1 NREVLFREPKRGIDEYLENDSFLQMIPVKYREIVLPKLRRDTNK---------------- 478

Yrf1-5 NREVLFREPKRGIDEYLENDSFLQMIPVKYREIVLPKLRRDTNK---------------- 478

Yrf1-8 NREVLFREPKRGIDEYLENDSFLQMIPVKYREIVLPKLRRDTNK---------------- 478

Yrf1-6 NREVLFREPKRGIDEYLENDSFLQMIPVKYREIVLPKLRRDTNK---------------- 542

Yrf1-7 NREVLFREPKRGIDEYLENDSFLQMIPVKYREIVLPKLRRDTNK---------------- 542

Yrf1-2 NREVLFREPKRGIDEYLENDSFLQMIPVKYREIVLPKLRRDTNK---------------- 364

Yrf1-3 NREVLFREPKRGIDEYLENDSFLQMIPVKYREIVLPKLRRDTNK---------------- 542

.***: . ***: *.: * *. : ** :* *

CaYrf1-5L SSIDRPYNDFVAHAKFDHQSYGLTMNMENNAYLKYDVARHASQKHAIVDLIDSMTRILYV 528

Yrf1-4 ----------------------MTA----------------ALKNKVAVAIDELTVPLMW 86

Yrf1-1 ----------------------MTA----------------ALKNKVTVAIDELTVPLMW 500

Yrf1-5 ----------------------MTA----------------ALKNKVTVAIDELTVPLMW 500

Yrf1-8 ----------------------MTA----------------ALKNKVTVAIDELTVPLMW 500

Yrf1-6 ----------------------MTA----------------ALKNKVTVAIDELTVPLMW 564

Yrf1-7 ----------------------MTA----------------ALKNKVTVAIDELTVPLMW 564

Yrf1-2 ----------------------MTA----------------ALKNKVTVAIDELTVPLMW 386

Yrf1-3 ----------------------MTA----------------ALKNKVTVAIDELTVPLMW 564

:* : *: :. **.:* *

CaYrf1-5L LIFTASANTFRATELSEVNVAANEVLNRNLIY--RHGAVEIVTSYGKNRKFTAR------ 580

Yrf1-4 MIHFAVGYPYRYPELQLLAFAGP---QRNVYVDDTTRRIQLYTDYNKNGSSEPRLKTLDG 143

Yrf1-1 MVHFAVGYPYRYPELQLLAFAGP---QRNVYVDDTTRRIQLYTDYNKNGSSEPRLKTLDG 557

Yrf1-5 MVHFAVGYPYRYPELQLLAFAGP---QRNVYVDDTTRRIQLYTDYNKNGSSEPRLKTLDG 557

Yrf1-8 MVHFAVGYPYRYPELQLLAFAGP---QRNVYVDDTTRRIQLYTDYNKNGSSEPRLKTLDG 557

Yrf1-6 MIHFAVGYPYRYPELQLLAFAGP---QRNVYVDDTTRRIQLYTDYNKNGSSEPRLKTLDG 621

Yrf1-7 MIHFAVGYPYRYPELQLLAFAGP---QRNVYVDDTTRRIQLYTDYNKNGSSEPRLKTLDG 621

Yrf1-2 MIHFAVGYPYRYPELQLLAFAGP---QRNVYVDDTTRRIQLYTDYNKNGSSEPRLKTLDG 443

Yrf1-3 MIHFAVGYPYRYPELQLLAFAGP---QRNVYVDDTTRRIQLYTDYNKNGSSEPRLKTLDG 621

::. * . :* **. : .*. :**: ::: *.*.** . *

CaYrf1-5L ----ERFYPLQLQKL-----------IVNHSLSMKPLLLRAMTGIGEMDAIHVNFHRYQL 625

Yrf1-4 LTSDYVFYFVTVLRQMQICALGNSYDAFNHDPWMDVVGFEDPDQVTNRDISRIVLYSYMF 203

Yrf1-1 LTSDYVFYFVTVLRQMQICALGNSYDAFNHDPWMDVVGFEDPDQVTNRDISRIVLYSYMF 617

Yrf1-5 LTSDYVFYFVTVLRQMQICALGNSYDAFNHDPWMDVVGFEDPDQVTNRDISRIVLYSYMF 617

Yrf1-8 LTSDYVFYFVTVLRQMQICALGNSYDAFNHDPWMDVVGFEDPDQVTNRDISRIVLYSYMF 617

Yrf1-6 LTSDYVFYFVTVLRQMQICALGNSYDAFNHDPWMDVVGFEDPDQVTNRDISRIVLYSYMF 681

Yrf1-7 LTSDYVFYFVTVLRQMQICALGNSYDAFNHDPWMDVVGFEDPDQVTNRDISRIVLYSYMF 681

Yrf1-2 LTSDYVFYFVTVLRQMQICALGNSYDAFNHDPWMDVVGFEDPDQVTNRDISRIVLYSYMF 503

Yrf1-3 LTSDYVFYFVTVLRQMQICALGNSYDAFNHDPWMDVVGFEDPDQVTNRDISRIVLYSYMF 681

** : : : .**. *. : :. : : * :: :: * :

CaYrf1-5L FALSSNKLVERGHYYSTCRTIMKDFG---GDIVRIREMRQAVSYFIKNAVVVDSKLDALE 682

Yrf1-4 LNTAKGCLVEY----ATFRQYMRELPKNAPQKLNFREMRQGLIALGRHCVGSRFETDLYE 259

Yrf1-1 LNTAKGCLVEY----ATFRQYMRELPKNAPQKLNFREMRQGLIALGRHCVGSRFETDLYE 673

Yrf1-5 LNTAKGCLVEY----ATFRQYMRELPKNAPQKLNFREMRQGLIALGRHCVGSRFETDLYE 673

Yrf1-8 LNTAKGCLVEY----ATFRQYMRELPKNAPQKLNFREMRQGLIALGRHCVGSRFETDLYE 673

Yrf1-6 LNTAKGCLVEY----ATFRQYMRELPKNAPQKLNFREMRQGLIALGRHCVGSRFETDLYE 737

Yrf1-7 LNTAKGCLVEY----ATFRQYMRELPKNAPQKLNFREMRQGLIALGRHCVGSRFETDLYE 737

Yrf1-2 LNTAKGCLVEY----ATFRQYMRELPKNAPQKLNFREMRQGLIALGRHCVGSRFETDLYE 559

Yrf1-3 LNTAKGCLVEY----ATFRQYMRELPKNAPQKLNFREMRQGLIALGRHCVGSRFETDLYE 737

: :.. *** :* * *::: : :.:*****.: : ::.* : * *

CaYrf1-5L SAVER-AQGYTMQTS------------------------------DESYAIENSWSCHFS 711

Yrf1-4 SATSELMANHSVQTGRNIYGVDSFSLTSVSGTTATLLQERASERWIQWLGLESDYHCSFS 319

Yrf1-1 SATSELMANHSVQTGRNIYGVDSFSLTSVSGTTATLLQERASERWIQWLGLESDYHCSFS 733

Yrf1-5 SATSELMANHSVQTGRNIYGVDSFSLTSVSGTTATLLQERASERWIQWLGLESDYHCSFS 733

Yrf1-8 SATSELMANHSVQTGRNIYGVDSFSLTSVSGTTATLLQERASERWIQWLGLESDYHCSFS 733

Yrf1-6 SATSELMANHSVQTGRNIYGVDSFSLTSVSGTTATLLQERASERWIQWLGLESDYHCSFS 797

Yrf1-7 SATSELMANHSVQTGRNIYGVDSFSLTSVSGTTATLLQERASERWIQWLGLESDYHCSFS 797

Yrf1-2 SATSELMANHSVQTGRNIYGVDSFSLTSVSGTTATLLQERASERWIQWLGLESDYHCSFS 619

Yrf1-3 SATSELMANHSVQTGRNIYGVDSFSLTSVSGTTATLLQERASERWIQWLGLESDYHCSFS 797

**... .:::**. : .:*..: * **

CaYrf1-5L SSNSQRMLKFFTEYHRFMRFDPIYSEVEYEKEEVASNSSNWRVLTTEYISQAKEI--CGY 769

Yrf1-4 STRNAEDVVAGEA--ASSDHHQKISRVTRKR-------PREPKSTNDILVAGRKLFGSSF 370

Yrf1-1 STRNAEDVVAGEA--ASSDHDQKISRVTRKR-------PREPKSTNDILVAGQKLFGSSF 784

Yrf1-5 STRNAEDVVAGEA--ASSDHDQKISRVTRKR-------PREPKSTNDILVAGQKLFGSSF 784

Yrf1-8 STRNAEDVVAGEA--ASSDHDQKISRVTRKR-------PREPKSTNDILVAGQKLFGSSF 784

Yrf1-6 STRNAEDVVAGEA--ASSDHHQKISRVTRKR-------PREPKSTNDILVAGQKLFGSSF 848

Yrf1-7 STRNAEDVVAGEA--ASSDHHQKISRVTRKR-------PREPKSTNDILVAGQKLFGSSF 848

Yrf1-2 STRNAEDVVAGEA--ASSDHHQKISRVTRKR-------PREPKSTNDILVAGQKLFGSSF 670

Yrf1-3 STRNAEDVVAGEA--ASSDHHQKISRVTRKR-------PREPKSTNDILVAGQKLFGSSF 848

*:.. . : .. *.* :: . *.: : .::: ..:

CaYrf1-5L SFRDLDQETAITDVAFTPTRSRAILAHTGFGKTFVFLVPMIAYKIGGGGEHFVHLVLMPY 829

Yrf1-4 EFRDLHQLRLCHEIYMADTPSVAVQAPPGYGKTELFHLPLIALASK-GDVKYVSFLFVPY 429

Yrf1-1 EFRDLHQLRLCHEIYMADTPSVAVQAPPGYGKTELFHLPLIALASK-GDVKYVSFLFVPY 843

Yrf1-5 EFRDLHQLRLCHEIYMADTPSVAVQAPPGYGKTELFHLPLIALASK-GDVKYVSFLFVPY 843

Yrf1-8 EFRDLHQLRLCHEIYMADTPSVAVQAPPGYGKTELFHLPLIALASK-GDVKYVSFLFVPY 843

Yrf1-6 EFRDLHQLRLCHEIYMADTPSVAVQAPPGYGKTELFHLPLIALASK-GDVKYVSFLFVPY 907

Yrf1-7 EFRDLHQLRLCHEIYMADTPSVAVQAPPGYGKTELFHLPLIALASK-GDVKYVSFLFVPY 907

Yrf1-2 EFRDLHQLRLCHEIYMADTPSVAVQAPPGYGKTELFHLPLIALASK-GDVKYVSFLFVPY 729

Yrf1-3 EFRDLHQLRLCHEIYMADTPSVAVQAPPGYGKTELFHLPLIALASK-GDVKYVSFLFVPY 907

.****.* :: :: * * *: * *:*** :* :*:** *. ::* ::::**

CaYrf1-5L RFLAEQMKTRLRRYLHV----IDAYEHRSFSDGVDVIVGVFDCLRNREFVNFILNFQNLP 885

Yrf1-4 TVLLANCMIRLSRCGCLNVAPVRNFIEEGCDGVTDLYVGIYDDLASTNFTDRIAAWENIV 489

Yrf1-1 TVLLANCMIRLSRCGCLNVAPVRNFIEEGCDGVTDLYVGIYDDLASTNFTDRIAAWENIV 903

Yrf1-5 TVLLANCMIRLSRCGCLNVAPVRNFIEEGCDGVTDLYVGIYDDLASTNFTDRIAAWENIV 903

Yrf1-8 TVLLANCMIRLSRCGCLNVAPVRNFIEEGCDGVTDLYVGIYDDLASTNFTDRIAAWENIV 903

Yrf1-6 TVLLANCMIRLSRCGCLNVAPVRNFIEEGCDGVTDLYVGIYDDLASTNFTDRIAAWENIV 967

Yrf1-7 TVLLANCMIRLSRCGCLNVAPVRNFIEEGCDGVTDLYVGIYDDLASTNFTDRIAAWENIV 967

Yrf1-2 TVLLANCMIRLSRCGCLNVAPVRNFIEEGCDGVTDLYVGIYDDLASTNFTDRIAAWENIV 789

Yrf1-3 TVLLANCMIRLSRCGCLNVAPVRNFIEEGCDGVTDLYVGIYDDLASTNFTDRIAAWENIV 967

.* : ** * : : : ... .. .*: **::* * . :*.: * ::*:

CaYrf1-5L -C---GRNSRLGMVVIDEAQVLNE-EYK---FRNFSNLRYECLKVFFKVVCLGATLGRD- 936

Yrf1-4 ECTFRTNNVKLGYLIVDEFHNFETEVYRQSQFGGITNLDFDAF---EKAIFLSGTAPEAV 546

Yrf1-1 ECTFRTNNVKLGYLIVDEFHNFETEVYRQSQFGGITNLDFDAF---EKAIFLSGTAPEAV 960

Yrf1-5 ECTFRTNNVKLGYLIVDEFHNFETEVYRQSQFGGITNLDFDAF---EKAIFLSGTAPEAV 960

Yrf1-8 ECTFRTNNVKLGYLIVDEFHNFETEVYRQSQFGGITNLDFDAF---EKAIFLSGTAPEAV 960

Yrf1-6 ECTFRTNNVKLGYLIVDEFHNFETEVYRQSQFGGITNLDFDAF---EKAIFLSGTAPEAV 1024

Yrf1-7 ECTFRTNNVKLGYLIVDEFHNFETEVYRQSQFGGITNLDFDAF---EKAIFLSGTAPEAV 1024

Yrf1-2 ECTFRTNNVKLGYLIVDEFHNFETEVYRQSQFGGITNLDFDAF---EKAIFLSGTAPEAV 846

Yrf1-3 ECTFRTNNVKLGYLIVDEFHNFETEVYRQSQFGGITNLDFDAF---EKAIFLSGTAPEAV 1024

* .* :** :::** : :: *: * .::** ::.: *.: *..* .

CaYrf1-5L ----FCRMNSRTLL----------------------SPVMMNCVRELPNCNVYMDQRVGD 970

Yrf1-4 ADAALQRIGLTGLAKKSMDINELKRSEDLSRGLSSYPTRMFNLIKEKSEV------PLGH 600

Yrf1-1 ADAALQRIGLTGLAKKSMDINELKRSEDLSRGLSSYPTRMFNLIKEKSEV------PLGH 1014

Yrf1-5 ADAALQRIGLTGLAKKSMDINELKRSEDLSRGLSSYPTRMFNLIKEKSEV------PLGH 1014

Yrf1-8 ADAALQRIGLTGLAKKSMDINELKRSEDLSRGLSSYPTRMFNLIKEKSEV------PLGH 1014

Yrf1-6 ADAALQRIGLTGLAKKSMDINELKRSEDLSRGLSSYPTRMFNLIKEKSEV------PLGH 1078

Yrf1-7 ADAALQRIGLTGLAKKSMDINELKRSEDLSRGLSSYPTRMFNLIKEKSEV------PLGH 1078

Yrf1-2 ADAALQRIGLTGLAKKSMDINELKRSEDLSRGLSSYPTRMFNLIKEKSEV------PLGH 900

Yrf1-3 ADAALQRIGLTGLAKKSMDINELKRSEDLSRGLSSYPTRMFNLIKEKSEV------PLGH 1078

: *:. * *:* ::* : :*.

CaYrf1-5L SRELMYVKLKQY-------VKNFVNYYPDD-----------LVLVYYDWKETLYAHEAEL 1012

Yrf1-4 VHK-IWKKVESQPEEALKLLLALFEIEPESKAIVVASTTNEVEELACSWRKYF------- 652

Yrf1-1 VHK-IWKKVESQPEEALKLLLALFEIEPESKAIVVASTTNEVEELACSWRKYF------- 1066

Yrf1-5 VHK-IWKKVESQPEEALKLLLALFEIEPESKAIVVASTTNEVEELACSWRKYF------- 1066

Yrf1-8 VHK-IWKKVESQPEEALKLLLALFEIEPESKAIVVASTTNEVEELACSWRKYF------- 1066

Yrf1-6 VHK-IWKKVESQPEEALKLLLALFEIEPESKAIVVASTTNEVEELACSWRKYF------- 1130

Yrf1-7 VHK-IWKKVESQPEEALKLLLALFEIEPESKAIVVASTTNEVEELACSWRKYF------- 1130

Yrf1-2 VHK-IWKKVESQPEEALKLLLALFEIEPESKAIVVASTTNEVEELACSWRKYF------- 952

Yrf1-3 VHK-IWKKVESQPEEALKLLLALFEIEPESKAIVVASTTNEVEELACSWRKYF------- 1130

:: :: *::. : :.: *:. : : .*:: :

CaYrf1-5L RRSYGDGVVTVTADIEDMEGVQDV--VRTAKVVLATKSFSCGIDLPNIRAIVFFDTDVPV 1070

Yrf1-4 RVVWIHGKLGAAE---KVSRTKEFVTDGSMRVLIGTKLVTEGIDIKQLMMVIMLDNRLNI 709

Yrf1-1 RVVWIHGKLGAAE---KVSRTKEFVTDGSMRVLIGTKLVTEGIDIKQLMMVIMLDNRLNI 1123

Yrf1-5 RVVWIHGKLGAAE---KVSRTKEFVTDGSMRVLIGTKLVTEGIDIKQLMMVIMLDNRLNI 1123

Yrf1-8 RVVWIHGKLGAAE---KVSRTKEFVTDGSMRVLIGTKLVTEGIDIKQLMMVIMLDNRLNI 1123

Yrf1-6 RVVWIHGKLGAAE---KVSRTKEFVTDGSMRVLIGTKLVTEGIDIKQLMMVIMLDNRLNI 1187

Yrf1-7 RVVWIHGKLGAAE---KVSRTKEFVTDGSMRVLIGTKLVTEGIDIKQLMMVIMLDNRLNI 1187

Yrf1-2 RVVWIHGKLGAAE---KVSRTKEFVTDGSMRVLIGTKLVTEGIDIKQLMMVIMLDNRLNI 1009

Yrf1-3 RVVWIHGKLGAAE---KVSRTKEFVTDGSMRVLIGTKLVTEGIDIKQLMMVIMLDNRLNI 1187

* : .* : .: .:. .::. : :*::.** .: ***: :: ::::*. : :

CaYrf1-5L AEMIQVIGRARNKVSHMVE----------LYSNRDPDEVRCFRQQMAAFYGIRAA----- 1115

Yrf1-4 IELIQGVGRLRDGGLCYLLSRKNSWAARNRKGELPPIKEGCITEQVREFYGLESKKGKKG 769

Yrf1-1 IELIQGVGRLRDGGLCYLLSRKNSWAARNRKGELPPIKEGCITEQVREFYGLESKKGKKG 1183

Yrf1-5 IELIQGVGRLRDGGLCYLLSRKNSWAARNRKGELPPIKEGCITEQVREFYGLESKKGKKG 1183

Yrf1-8 IELIQGVGRLRDGGLCYLLSRKNSWAARNRKGELPPIKEGCITEQVREFYGLESKKGKKG 1183

Yrf1-6 IELIQGVGRLRDGGLCYLLSRKNSWAARNRKGELPPIKEGCITEQVREFYGLESKKGKKG 1247

Yrf1-7 IELIQGVGRLRDGGLCYLLSRKNSWAARNRKGELPPIKEGCITEQVREFYGLESKKGKKG 1247

Yrf1-2 IELIQGVGRLRDGGLCYLLSRKNSWAARNRKGELPPIKEGCITEQVREFYGLESKKGKKG 1069

Yrf1-3 IELIQGVGRLRDGGLCYLLSRKNSWAARNRKGELPPIKEGCITEQVREFYGLESKKGKKG 1247

*:** :** *: : .: * : *: :*: ***:.:

CaYrf1-5L -CNNCCGVVDAEHEDMLRKLWEDEP---EETNQYDEIVVVAETEEEKNWYDEIVDEAESI 1171

Yrf1-4 QHVGCCGSRTDLSADTVELIERMDRLAEKQATASMSIIALPSSFQESNSS---------- 819

Yrf1-1 QHVGCCGSRTDLSADTVELIERMDRLAEKQATASMSIIALPSSFQESNSS---------- 1233

Yrf1-5 QHVGCCGSRTDLSADTVELIERMDRLAEKQATASMSIIALPSSFQESNSS---------- 1233

Yrf1-8 QHVGCCGSRTDLSADTVELIERMDRLAEKQATASMSIIALPSSFQESNSS---------- 1233

Yrf1-6 QHVGCCGSRTDLSADTVELIERMDRLAEKQATASMSIVALPSSFQESNSS---------- 1297

Yrf1-7 QHVGCCGSRTDLSADTVELIERMDRLAEKQATASMSIVALPSSFQESNSS---------- 1297

Yrf1-2 QHVGCCGSRTDLSADTVELIERMDRLAEKQATASMSIVALPSSFQESNSS---------- 1119

Yrf1-3 QHVGCCGSRTDLSADTVELIERMDRLAEKQATASMSIVALPSSFQESNSS---------- 1297

.*** * :. : . : :::. .*:.: .: :*.*

CaYrf1-5L MQQIEMGNPVIGSTFKYCKMAKYIQMF----------------------------KSQPT 1203

Yrf1-4 -----------DRCRKYCSSDEDSDTCIHGSANASTNATTNSSTNATTTASTNVRTSATT 868

Yrf1-1 -----------DRCRKYCSSDEDSDTCIHGSANASTNATTNSSTNATTTASTNVRTSATT 1282

Yrf1-5 -----------DRCRKYCSSDEDSDTCIHGSANASTNATTNSSTNATTTASTNVRTSATT 1282

Yrf1-8 -----------DRCRKYCSSDEDSDTCIHGSANASTNATTNSSTNATTTASTNVRTSATT 1282

Yrf1-6 -----------DRCRKYCSSDEDSDTCIHGSANASTNATTNSSTNATTTASTNVRTSATT 1346

Yrf1-7 -----------DRCRKYCSSDEDSDTCIHGSANASTNATTNSSTNATTTASTNVRTSATT 1346

Yrf1-2 -----------DRCRKYCSSDEDSDTCIHGSANASTNATTNSSTNATTTASTNVRTSATT 1168

Yrf1-3 -----------DRCRKYCSSDEDSDTCIHGSANASTNATTNSSTNATTTASTNVRTSATT 1346

. ***. : : .* *

CaYrf1-5L FEHVDMCGYCYMPVAYCTRGRHGCRYQK--TVKNVMMVRWILGDIDVEGIKEIIGGVEGV 1261

Yrf1-4 TASINVR-----TSAITTESTNSSTNATTTASTNVRTSATTTASINVRTSATTTESTNSN 923

Yrf1-1 TASINVR-----TSAITTESTNSSTNATTTASTNVRTSATTTASINVRTSATTTESTNSN 1337

Yrf1-5 TASINVR-----TSAITTESTNSSTNATTTASTNVRTSATTTASINVRTSATTTESTNSN 1337

Yrf1-8 TASINVR-----TSAITTESTNSSTNATTTASTNVRTSATTTASINVRTSATTTESTNSN 1337

Yrf1-6 TASINVR-----TSATTTESTNSSTNATTTASTNVRTSATTTASINVRTSATTTESTNSN 1401

Yrf1-7 TASINVR-----TSATTTESTNSSTNATTTASTNVRTSATTTASINVRTSATTTESTNSN 1401

Yrf1-2 TASINVR-----TSATTTESTNSSTNATTTASTNVRTSATTTASINVRTSATTTESTNSN 1223

Yrf1-3 TASINVR-----TSATTTESTNSSTNATTTASTNVRTSATTTASINVRTSATTTESTNSN 1401

::: * *.. :.. . : .** ..*:*. ..:.

CaYrf1-5L EQINWIRENEEYASAGIREYCQR------------------------------------- 1284

Yrf1-4 TSATTTESTDSNTSATTTESTDSNTSATTTASTNSSTNATTTASTNSSTNATTTESTNAS 983

Yrf1-1 TSATTTESTDSNTSATTTESTDSNTSATTTASTNSSTNATTTASTNSSTNATTTESTNAS 1397

Yrf1-5 TSATTTESTDSNTSATTTESTDSNTSATTTASTNSSTNATTTASTNSSTNATTTESTNAS 1397

Yrf1-8 TSATTTESTDSNTSATTTESTDSNTSATTTASTNSSTNATTTASTNSSTNATTTESTNAS 1397

Yrf1-6 TSATTTESTDSNTSATTTESTDSNTSATTTASTNSSTNATTTASTNSSTNATTTESTNAS 1461

Yrf1-7 TSATTTESTDSNTSATTTESTDSNTSATTTASTNSSTNATTTASTNSSTNATTTESTNAS 1461

Yrf1-2 TSATTTESTDSNTSATTTESTDSNTSATTTASTNSSTNATTTASTNSSTNATTTESTNAS 1283

Yrf1-3 TSATTTESTDSNTSATTTESTDSNTSATTTASTNSSTNATTTASTNSSTNATTTESTNAS 1461

. . ...:. :** * :

CaYrf1-5L ----------MVSHSFINVDELPRGMPTTQSMFLMVVEQWKDRSVFGEGEEDQAVARKAD 1334

Yrf1-4 AKEDANKDGNAEDNRFHPVTDINKESYKRKGSQMVLLERKKLKAQFPNTSEN-------- 1035

Yrf1-1 AKEDANKDGNAEDNRFHPVTDINKESYKRKGSQMVLLERKKLKAQFPNTSEN-------- 1449

Yrf1-5 AKEDANKDGNAEDNRFHPVTDINKESYKRKGSQMVLLERKKLKAQFPNTSEN-------- 1449

Yrf1-8 AKEDANKDGNAEDNRFHPVTDINKESYKRKGSQMVLLERKKLKAQFPNTSEN-------- 1449

Yrf1-6 AKEDANKDGNAEDNRFHPVTDINKESYKRKGSQMVLLERKKLKAQFPNTSEN-------- 1513

Yrf1-7 AKEDANKDGNAEDNRFHPVTDINKESYKRKGSQMVLLERKKLKAQFPNTSEN-------- 1513

Yrf1-2 AKEDANKDGNAEDNRFHPVTDINKESYKRKGSQMVLLERKKLKAQFPNTSEN-------- 1335

Yrf1-3 AKEDANKDGNAEDNRFHPVTDINKESYKRKGSQMVLLERKKLKAQFPNTSEN-------- 1513

.: * * :: : . :. ::::*: * :: * : .*:

CaYrf1-5L GYFASNWRNEVEYMTFKMED---------RCKWCGVGVGR----HQCCGRFI-------- 1373

Yrf1-4 -------MNVLQFLGFRSDEIKHLFLYGIDVYFCPEGVFTQYGLCKGCQKMFELCVCWAG 1088

Yrf1-1 -------MNVLQFLGFRSDEIKHLFLYGIDVYFCPEGVFTQYGLCKGCQKMFELCVCWAG 1502

Yrf1-5 -------MNVLQFLGFRSDEIKHLFLYGIDVYFCPEGVFTQYGLCKGCQKMFELCVCWAG 1502

Yrf1-8 -------MNVLQFLGFRSDEIKHLFLYGIDVYFCPEGVFTQYGLCKGCQKMFELCVCWAG 1502

Yrf1-6 -------MNVLQFLGFRSDEIKHLFLYGIDVYFCPEGVFTQYGLCKGCQKMFELCVCWAG 1566

Yrf1-7 -------MNVLQFLGFRSDEIKHLFLYGIDVYFCPEGVFTQYGLCKGCQKMFELCVCWAG 1566

Yrf1-2 -------MNVLQFLGFRSDEIKHLFLYGIDVYFCPEGVFTQYGLCKGCQKMFELCVCWAG 1388

Yrf1-3 -------MNVLQFLGFRSDEIKHLFLYGIDVYFCPEGVFTQYGLCKGCQKMFELCVCWAG 1566

* :::: *: :: :* ** : * :::

CaYrf1-5L -----KKLLYAIF-------VDERMRGYV-------GDMRIFEHWFEAVGVGEVFMRLVI 1414

Yrf1-4 QKVSYRRMAWEALAVERMLRNDEEYKEYLEDIEPYHGDPVGYLKYF-SVKRGEIYSQIQR 1147

Yrf1-1 QKVSYRRMAWEALAVERMLRNDEEYKEYLEDIEPYHGDPVGYLKYF-SVKRGEIYSQIQR 1561

Yrf1-5 QKVSYRRMAWEALAVERMLRNDEEYKEYLEDIEPYHGDPVGYLKYF-SVKRGEIYSQIQR 1561

Yrf1-8 QKVSYRRMAWEALAVERMLRNDEEYKEYLEDIEPYHGDPVGYLKYF-SVKRGEIYSQIQR 1561

Yrf1-6 QKVSYRRMAWEALAVERMLRNDEEYKEYLEDIEPYHGDPVGYLKFF-SVKRGEIYSQIQR 1625

Yrf1-7 QKVSYRRMAWEALAVERMLRNDEEYKEYLEDIEPYHGDPVGYLKYF-SVKRGEIYSQIQR 1625

Yrf1-2 QKVSYRRMAWEALAVERMLRNDEEYKEYLEDIEPYHGDPVGYLKYF-SVKRGEIYSQIQR 1447

Yrf1-3 QKVSYRRMAWEALAVERMLRNDEEYKEYLEDIEPYHGDPVGYLKYF-SVKRGEIYSQIQR 1625

::: : : **. : *: ** : ::* :* **:: ::

CaYrf1-5L RY--YEVVTE-------------------------------------------------- 1422

Yrf1-4 NYAWYLAITRRRETISVLDSTRGKQGSQVFRMSGRQIKELYYKVWSNLRESKTEVLQYFL 1207

Yrf1-1 NYAWYLAITRRRETISVLDSTRGKQGSQVFRMSGRQIKELYYKVWSNLRESKTEVLQYFL 1621

Yrf1-5 NYAWYLAITRRRETISVLDSTRGKQGSQVFRMSGRQIKELYYKVWSNLRESKTEVLQYFL 1621

Yrf1-8 NYAWYLAITRRRETISVLDSTRGKQGSQVFRMSGRQIKELYYKVWSNLRESKTEVLQYFL 1621

Yrf1-6 NYAWYLAITRRRETISVLDSTRGKQGSQVFRMSGRQIKELYYKVWSNLRESKTEVLQYFL 1685

Yrf1-7 NYAWYLAITRRRETISVLDSTRGKQGSQVFRMSGRQIKELYYKVWSNLRESKTEVLQYFL 1685

Yrf1-2 NYAWYLAITRRRETISVLDSTRGKQGSQVFRMSGRQIKELYYKVWSNLRESKTEVLQYFL 1507

Yrf1-3 NYAWYLAITRRRETISVLDSTRGKQGSQVFRMSGRQIKELYYKVWSNLRESKTEVLQYFL 1685

.* * .:*.

CaYrf1-5L ------------------------------------------------------------ 1422

Yrf1-4 NWDEKKCREEWEAKDDTVFVEALEKVGVFQRLRSMTSAGLQGPQYVKLQFSRHHRQLRSR 1267

Yrf1-1 NWDEKKCREEWEAKDDTVFVEALEKVGVFQRLRSMTSAGLQGPQYVKLQFSRHHRQLRSR 1681

Yrf1-5 NWDEKKCREEWEAKDDTVFVEALEKVGVFQRLRSMTSAGLQGPQYVKLQFSRHHRQLRSR 1681

Yrf1-8 NWDEKKCREEWEAKDDTVFVEALEKVGVFQRLRSMTSAGLQGPQYVKLQFSRHHRQLRSR 1681

Yrf1-6 NWDEKKCREEWEAKDDTVFVEALEKVGVFQRLRSMTSAGLQGPQYVKLQFSRHHRQLRSR 1745

Yrf1-7 NWDEKKCREEWEAKDDTVFVEALEKVGVFQRLRSMTSAGLQGPQYVKLQFSRHHRQLRSR 1745

Yrf1-2 NWDEKKCREEWEAKDDTVFVEALEKVGVFQRLRSMTSAGLQGPQYVKLQFSRHHRQLRSR 1567

Yrf1-3 NWDEKKCREEWEAKDDTVFVEALEKVGVFQRLRSMTSAGLQGPQYVKLQFSRHHRQLRSR 1745

CaYrf1-5L ------------------------------------------------------------ 1422

Yrf1-4 YELSLGMHLRDQLALGVTPSKVPHWTAFLSMLIGLFYNKTFRQKLEYLLEQISEVWLLPH 1327

Yrf1-1 YELSLGMHLRDQLALGVTPSKVPHWTAFLSMLIGLFYNKTFRQKLEYLLEQISEVWLLPH 1741

Yrf1-5 YELSLGMHLRDQLALGVTPSKVPHWTAFLSMLIGLFYNKTFRQKLEYLLEQISEVWLLPH 1741

Yrf1-8 YELSLGMHLRDQLALGVTPSKVPHWTAFLSMLIGLFYNKTFRQKLEYLLEQISEVWLLPH 1741

Yrf1-6 YELSLGMHLRDQLALGVTPSKVPHWTAFLSMLIGLFCNKTFRQKLEYLLEQISEVWLLPH 1805

Yrf1-7 YELSLGMHLRDQLALGVTPSKVPHWTAFLSMLIGLFCNKTFRQKLEYLLEQISEVWLLPH 1805

Yrf1-2 YELSLGMHLRDQLALGVTPSKVPHWTAFLSMLIGLFCNKTFRQKLEYLLEQISEVWLLPH 1627

Yrf1-3 YELSLGMHLRDQLALGVTPSKVPHWTAFLSMLIGLFCNKTFRQKLEYLLEQISEVWLLPH 1805

CaYrf1-5L ------------------------------------------------------- 1422

Yrf1-4 WLDLANVEVLAADNTRVPLYMLMVAVHKELDSDDVPDGRFDIILLCRDSSREVGE 1382

Yrf1-1 WLDLANVEVLAADNTRVPLYMLMVAVHKELDSDDVPDGRFDIILLCRDSSREVGE 1796

Yrf1-5 WLDLANVEVLAADNTRVPLYMLMVAVHKELDSDDVPDGRFDIILLCRDSSREVGE 1796

Yrf1-8 WLDLANVEVLAADNTRVPLYMLMVAVHKELDSDDVPDGRFDIILLCRDSSREVGE 1796

Yrf1-6 WLDLANVEVLAADNTRVPLYMLMVAVHKELDSDDVPDGRFDI-LLCRDSSREVGE 1859

Yrf1-7 WLDLANVEVLAADNTRVPLYMLMVAVHKELDSDDVPDGRFDI-LLCRDSSREVGE 1859

Yrf1-2 WLDLANVEVLAADNTRVPLYMLMVAVHKELDSDDVPDGRFDI-LLCRDSSREVGE 1681

Yrf1-3 WLDLANVEVLAADNTRVPLYMLMVAVHKELDSDDVPDGRFDI-LLCRDSSREVGE 1859

**SUPPLEMENTARY FIGURE S2 |** Clustal Omega (Madeira et al., 2019) alignment of amino acid sequences predicted by *C. albicans* *YRF1-5L* and the eight *S. cerevisiae YRF1* alleles. *C. albicans YRF1* alleles were nearly 100% identical so *YRF1-5L* was chosen as a representative sequence. Variations among *S. cerevisiae YRF1* alleles predicted proteins that were as much as 477 amino acids different in length. All were included in this diagram to illustrate products from the different translational start sites.
