# Supplementary material for: Use of a Candida albicans SC5314 PacBio HiFi reads dataset to close gaps in the reference genome assembly, reveal a subtelomeric gene family, and produce accurate phased allelic sequences
Source: Front Cell Infect Microbiol. 2024 Feb 1;14:1329438. doi: 10.3389/fcimb.2024.1329438 (PMC10867151; doi:10.3389/fcimb.2024.1329438)
Supplement: Supplementary file 2 [file DataSheet_2.docx]

**SUPPLEMENTARY FILE S1 |** Resources and commands needed to view the PacBio HiFi data set using the Integrative Genomics Viewer (IGV).

**1. Download data files**

**a).** PacBio HiFi Reads Data Set: <https://www.ncbi.nlm.nih.gov/sra/SRR23724250>

Download Run SRR23724250

· The example file name used below is SRR23724250.gz, representing a gzipped fastq dataset that contains all PacBio HiFi reads that were derived in the context of this study.

**b).** New *C. albicans* SC5314 Assembly: <https://www.ncbi.nlm.nih.gov/datasets/genome/GCA_032688725.1>

Download the FASTA genome sequence (GCA_032688725.1_ASM3268872v1_genomic.fna)

· The example file name used below is ASM3268872.fna representing a fasta dataset that contains the sequences for the eight *C. albicans* SC5314 chromosomes.

· This webpage also allows BLAST of the genome assembly. Use BLAST to locate your region of interest in the context of ASM3268872v1.

**2. Download analysis programs; versions used in the current study are listed**

a). filtlong v0.2.1

· <https://github.com/rrwick/Filtlong>

b). minimap2 v2.21

· <https://github.com/lh3/minimap2/releases>

c). SAMtools v1.12

· <https://sourceforge.net/projects/samtools/files/samtools/1.12>

d). Integrative Genomics Viewer v2.16.2

· <https://igv.org>

**3. Create the files needed to visualize read alignments with IGV**

· The HiFi reads data set is very large. The alignment on the Integrative Genomics Viewer will be simplified by only including the longest reads. The longest reads are most useful for assembling regions of extreme allelic heterogeneity.

· This filtlong command selects the reads that are at least20 kb from the PacBio HiFi reads data set (SRR23724250.gz). It zips them into a file (SRR23724250Filt.gz) that is used in the subsequent steps.

filtlong --min_length 20000 SRR23724250.gz | gzip > SRR23724250Filt.gz

· Minimap2 and SAMtools are used to create an unsorted bam file, then sort the bam file, and finally index the bam file. At the end of these steps, you will have both the bam and bam.bai files you need for IGV.

minimap2 -ax map-hifi ASM3268872.fna SRR23724250Filt.gz | samtools view -b - > Hifi_reads.bam

samtools sort Hifi_reads.bam -o Hifi_reads_sorted.bam

samtools index Hifi_reads_sorted.bam

**4. Load files into IGV**

· Place the genome assembly (ASM3268872.fna), bam (Hifi_reads_sorted.bam) and index file (Hifi_reads_sorted.bam.bai) in the same folder before proceeding to IGV.

· Open the IGV application.

· Under the Genomes tab, select “Load Genome from File” and direct the program to the ASM3268872.fna file.

· Under the File tab, choose “Load from File” and direct the program to the Hifi_reads_sorted.bam file. The screen should now show the names of the 8 *C. albicans* chromosome sequences across the top of the screen. The bam file name should be visible in the panel at the left of the browser.

· Click on a chromosome and zoom in using the control at the top right of the browser. Zooming reveals details, down to the nucleotide sequence. Individual reads are indicated by gray bars, each showing the direction of the read relative to the genome assembly. Read coverage is visible near the top of the diagram. Gray is used to indicate sequence matches vertically. Insertions (blue I) are shown, as well as sequence variations coded to indicate the different nucleotides (A = green; C = blue; G = orange; T = red). Clicking on a read reveals its name and detailed information. Reads can be extracted from the SRR23724250Filt.gz file or the genome region of interest assembled from the IGV screen. To learn more about available features, consult the IGV manual at <https://igv.org>.
