# Supplementary material for: Use of a Candida albicans SC5314 PacBio HiFi reads dataset to close gaps in the reference genome assembly, reveal a subtelomeric gene family, and produce accurate phased allelic sequences
Source: Front Cell Infect Microbiol. 2024 Feb 1;14:1329438. doi: 10.3389/fcimb.2024.1329438 (PMC10867151; doi:10.3389/fcimb.2024.1329438)
Supplement: Supplementary file 3 [file Presentation_1.pptx]

## Slide 1
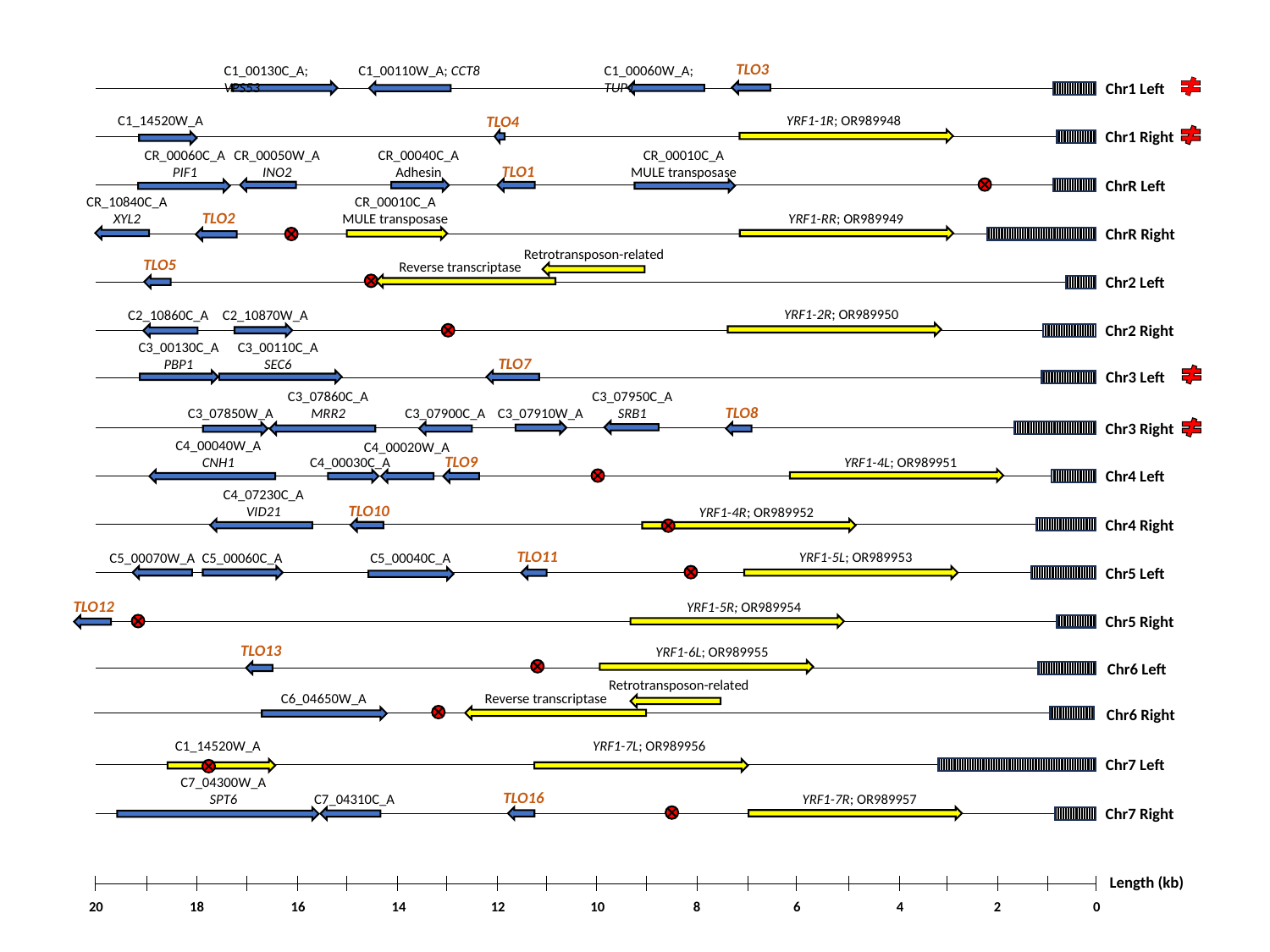

TLO3
C1_00130C_A; VPS53
C1_00110W_A; CCT8
C1_00060W_A; TUP1
Chr1 Left
C1_14520W_A
TLO4
YRF1-1R; OR989948
Chr1 Right
CR_00060C_A
PIF1
CR_00050W_A
INO2
CR_00040C_A
Adhesin
CR_00010C_A
MULE transposase
TLO1
ChrR Left
CR_10840C_A
XYL2
CR_00010C_A
MULE transposase
TLO2
YRF1-RR; OR989949
ChrR Right
Retrotransposon-related
TLO5
Reverse transcriptase
Chr2 Left
C2_10860C_A
C2_10870W_A
YRF1-2R; OR989950
Chr2 Right
C3_00130C_A
PBP1
C3_00110C_A
SEC6
Chr3 Left
TLO7
C3_07860C_A
MRR2
C3_07950C_A
SRB1
TLO8
C3_07850W_A
C3_07900C_A
C3_07910W_A
Chr3 Right
C4_00040W_A
CNH1
C4_00020W_A
TLO9
C4_00030C_A
YRF1-4L; OR989951
Chr4 Left
C4_07230C_A
VID21
TLO10
YRF1-4R; OR989952
Chr4 Right
TLO11
C5_00070W_A
C5_00060C_A
C5_00040C_A
YRF1-5L; OR989953
Chr5 Left
TLO12
YRF1-5R; OR989954
Chr5 Right
TLO13
YRF1-6L; OR989955
Chr6 Left
Retrotransposon-related
Reverse transcriptase
C6_04650W_A
Chr6 Right
C1_14520W_A
YRF1-7L; OR989956
Chr7 Left
C7_04300W_A
SPT6
TLO16
C7_04310C_A
YRF1-7R; OR989957
Chr7 Right
Length (kb)
20
18
16
14
12
10
8
6
4
2
0

## Slide 2
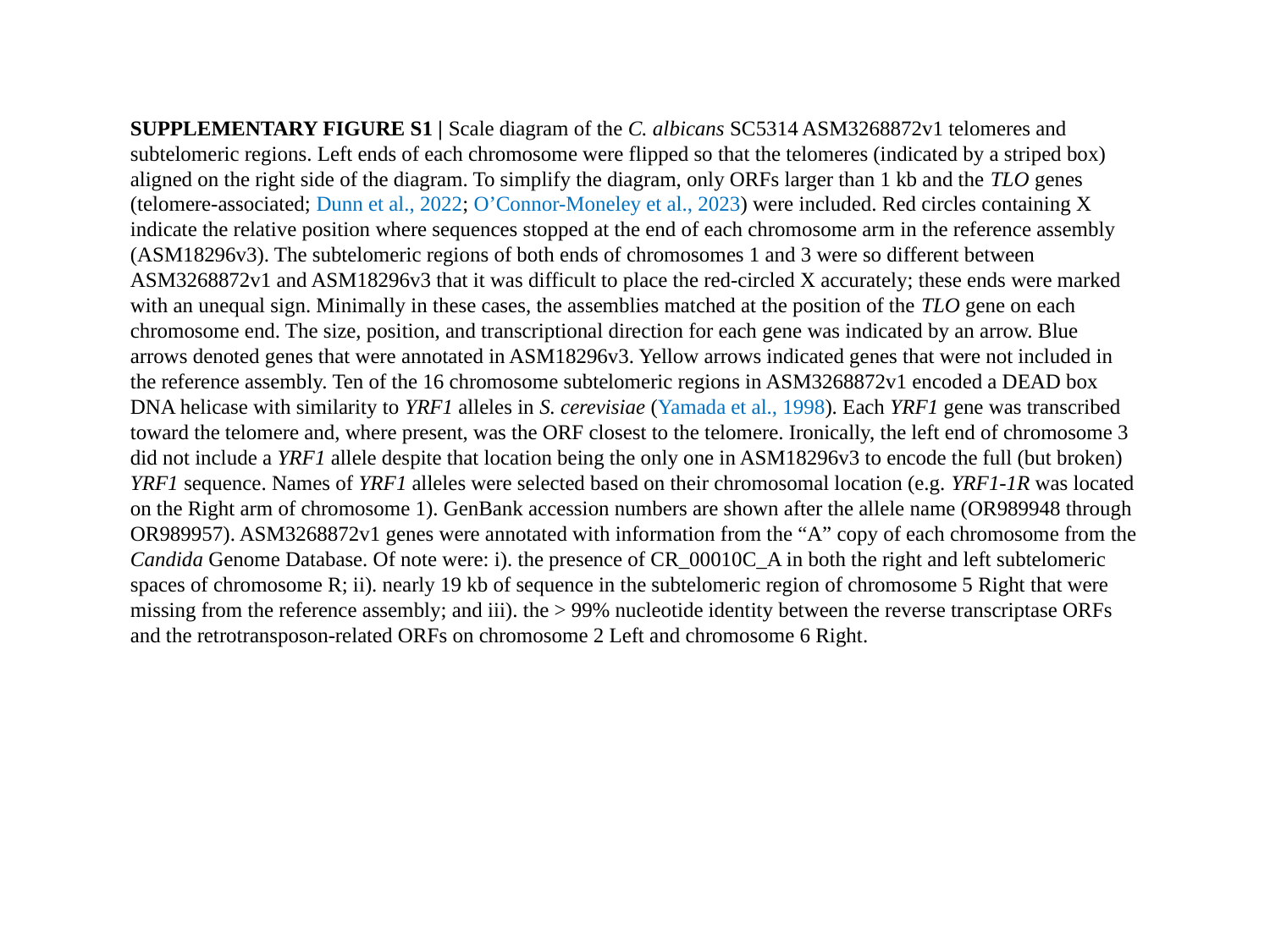

SUPPLEMENTARY FIGURE S1 | Scale diagram of the C. albicans SC5314 ASM3268872v1 telomeres and subtelomeric regions. Left ends of each chromosome were flipped so that the telomeres (indicated by a striped box) aligned on the right side of the diagram. To simplify the diagram, only ORFs larger than 1 kb and the TLO genes (telomere-associated; Dunn et al., 2022; O’Connor-Moneley et al., 2023) were included. Red circles containing X indicate the relative position where sequences stopped at the end of each chromosome arm in the reference assembly (ASM18296v3). The subtelomeric regions of both ends of chromosomes 1 and 3 were so different between ASM3268872v1 and ASM18296v3 that it was difficult to place the red-circled X accurately; these ends were marked with an unequal sign. Minimally in these cases, the assemblies matched at the position of the TLO gene on each chromosome end. The size, position, and transcriptional direction for each gene was indicated by an arrow. Blue arrows denoted genes that were annotated in ASM18296v3. Yellow arrows indicated genes that were not included in the reference assembly. Ten of the 16 chromosome subtelomeric regions in ASM3268872v1 encoded a DEAD box DNA helicase with similarity to YRF1 alleles in S. cerevisiae (Yamada et al., 1998). Each YRF1 gene was transcribed toward the telomere and, where present, was the ORF closest to the telomere. Ironically, the left end of chromosome 3 did not include a YRF1 allele despite that location being the only one in ASM18296v3 to encode the full (but broken) YRF1 sequence. Names of YRF1 alleles were selected based on their chromosomal location (e.g. YRF1-1R was located on the Right arm of chromosome 1). GenBank accession numbers are shown after the allele name (OR989948 through OR989957). ASM3268872v1 genes were annotated with information from the “A” copy of each chromosome from the Candida Genome Database. Of note were: i). the presence of CR_00010C_A in both the right and left subtelomeric spaces of chromosome R; ii). nearly 19 kb of sequence in the subtelomeric region of chromosome 5 Right that were missing from the reference assembly; and iii). the > 99% nucleotide identity between the reverse transcriptase ORFs and the retrotransposon-related ORFs on chromosome 2 Left and chromosome 6 Right.
