# Supplementary material for: Use of a Candida albicans SC5314 PacBio HiFi reads dataset to close gaps in the reference genome assembly, reveal a subtelomeric gene family, and produce accurate phased allelic sequences
Source: Front Cell Infect Microbiol. 2024 Feb 1;14:1329438. doi: 10.3389/fcimb.2024.1329438 (PMC10867151; doi:10.3389/fcimb.2024.1329438)
Supplement: Supplementary file 4 [file Presentation_2.pptx]

## Slide 1
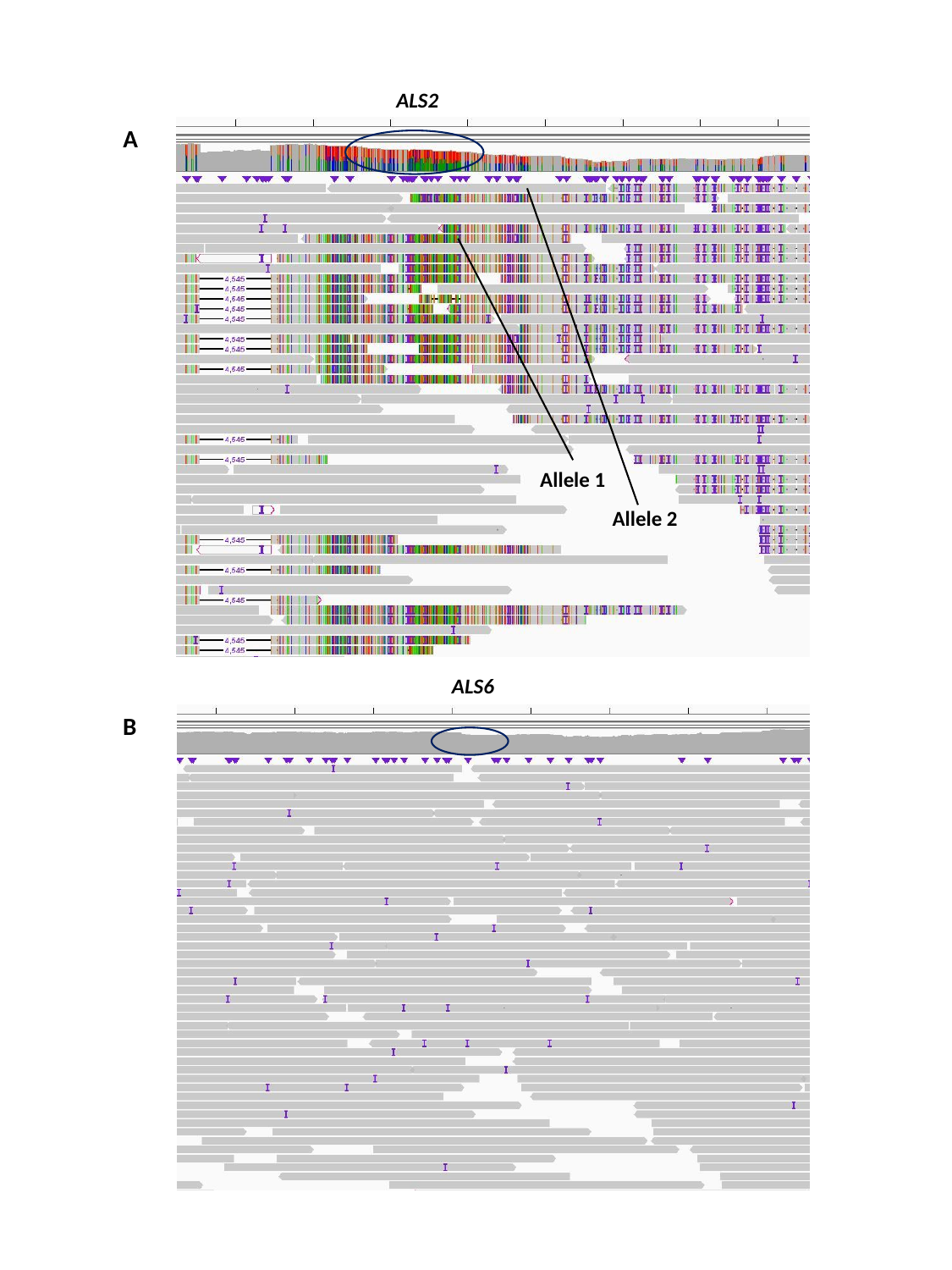

ALS2
A
Allele 1
Allele 2
ALS6
B

## Slide 2
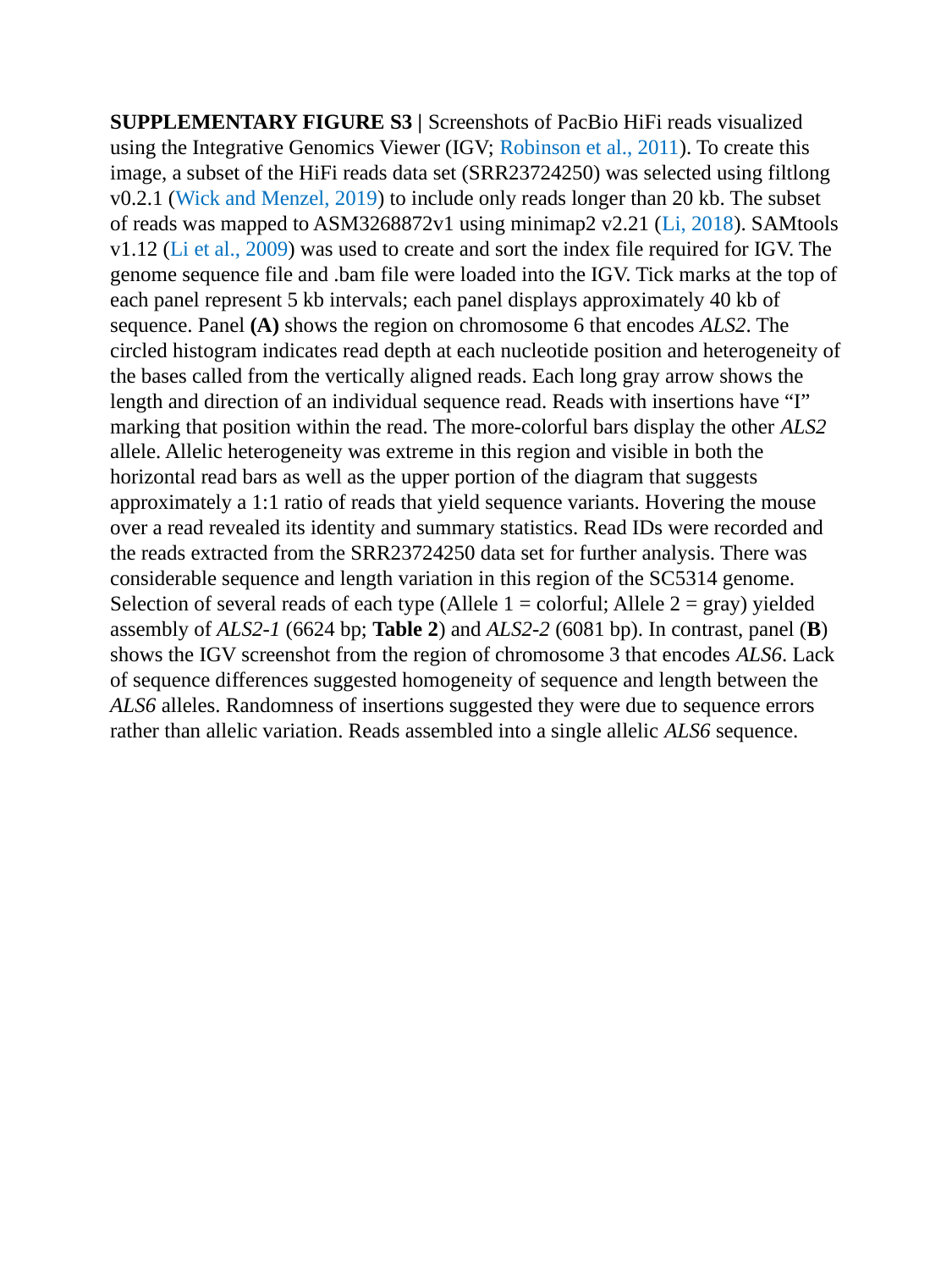

SUPPLEMENTARY FIGURE S3 | Screenshots of PacBio HiFi reads visualized using the Integrative Genomics Viewer (IGV; Robinson et al., 2011). To create this image, a subset of the HiFi reads data set (SRR23724250) was selected using filtlong v0.2.1 (Wick and Menzel, 2019) to include only reads longer than 20 kb. The subset of reads was mapped to ASM3268872v1 using minimap2 v2.21 (Li, 2018). SAMtools v1.12 (Li et al., 2009) was used to create and sort the index file required for IGV. The genome sequence file and .bam file were loaded into the IGV. Tick marks at the top of each panel represent 5 kb intervals; each panel displays approximately 40 kb of sequence. Panel (A) shows the region on chromosome 6 that encodes ALS2. The circled histogram indicates read depth at each nucleotide position and heterogeneity of the bases called from the vertically aligned reads. Each long gray arrow shows the length and direction of an individual sequence read. Reads with insertions have “I” marking that position within the read. The more-colorful bars display the other ALS2 allele. Allelic heterogeneity was extreme in this region and visible in both the horizontal read bars as well as the upper portion of the diagram that suggests approximately a 1:1 ratio of reads that yield sequence variants. Hovering the mouse over a read revealed its identity and summary statistics. Read IDs were recorded and the reads extracted from the SRR23724250 data set for further analysis. There was considerable sequence and length variation in this region of the SC5314 genome. Selection of several reads of each type (Allele 1 = colorful; Allele 2 = gray) yielded assembly of ALS2-1 (6624 bp; Table 2) and ALS2-2 (6081 bp). In contrast, panel (B) shows the IGV screenshot from the region of chromosome 3 that encodes ALS6. Lack of sequence differences suggested homogeneity of sequence and length between the ALS6 alleles. Randomness of insertions suggested they were due to sequence errors rather than allelic variation. Reads assembled into a single allelic ALS6 sequence.
